# The Composition and Function of Microbiomes Within *Microcystis* Colonies Are Significantly Different Than Native Bacterial Assemblages in Two North American Lakes

**DOI:** 10.3389/fmicb.2020.01016

**Published:** 2020-05-28

**Authors:** Jennifer G. Jankowiak, Christopher J. Gobler

**Affiliations:** School of Marine and Atmospheric Sciences, Stony Brook University, Southampton, NY, United States

**Keywords:** *Microcystis*, associated bacteria, next generation sequencing, microbiome, free-living, eutrophication

## Abstract

The toxic cyanobacterium *Microcystis* is one of the most pervasive harmful algal bloom (HAB) genera and naturally occurs in large colonies known to harbor diverse heterotrophic bacterial assemblages. While colony-associated microbiomes may influence *Microcystis* blooms, there remains a limited understanding of the structure and functional potential of these communities and how they may be shaped by changing environmental conditions. To address this gap, we compared the dynamics of *Microcystis*-attached (MCA), free-living (FL), and whole water (W) microbiomes during *Microcystis* blooms using next-generation amplicon sequencing (16S rRNA), a predictive metagenome software, and other bioinformatic approaches. Microbiomes were monitored through high resolution spatial-temporal surveys across two North American lakes, Lake Erie (LE) and Lake Agawam (LA; Long Island, NY, United States) in 2017, providing the largest dataset of these fractions to date. Sequencing of 126 samples generated 7,922,628 sequences that clustered into 7,447 amplicon sequence variants (ASVs) with 100% sequence identity. Across lakes, the MCA microbiomes were significantly different than the FL and W fractions being significantly enriched in *Gemmatimonadetes*, *Burkholderiaceae*, *Rhizobiales*, and *Cytophagales* and depleted of *Actinobacteria.* Further, although MCA communities harbored > 900 unique ASVs, they were significantly less diverse than the other fractions with diversity inversely related to bloom intensity, suggesting increased selection pressure on microbial communities as blooms intensified. Despite taxonomic differences between lakes, predicted metagenomes revealed conserved functional potential among MCA microbiomes. MCA communities were significantly enriched in pathways involved in N and P cycling and microcystin-degradation. Taxa potentially capable of N_2_-fixation were significantly enriched (*p* < 0.05) and up to four-fold more abundant within the MCA faction relative to other fractions, potentially aiding in the proliferation of *Microcystis* blooms during low N conditions. The MCA predicted metagenomes were conserved over 8 months of seasonal changes in temperature and N availability despite strong temporal succession in microbiome composition. Collectively, these findings indicate that *Microcystis* colonies harbor a statistically distinct microbiome with a conserved functional potential that may help facilitate bloom persistence under environmentally unfavorable conditions.

## Introduction

In recent decades the frequency, intensity and duration of harmful cyanobacteria blooms (CyanoHABs) in freshwater environments has expanded on a global scale (Carmichael, 2008; Stumpf et al., 2012; Huisman et al., 2018). Among the most prevalent bloom-forming cyanobacteria is the genus *Microcystis* (Zurawell et al., 2005; Harke et al., 2016) that can lead to ecosystem disruption through shading (Scheffer et al., 1993) and oxygen depletion (Rabalais et al., 2010; Paerl and Otten, 2013) and represent a public health threat via the production of the hepatotoxin, microcystin (Carmichael, 2001; Rantala et al., 2006; Merel et al., 2013). Among abiotic drivers of *Microcystis* blooms, there is a wealth of evidence that anthropogenic nitrogen (N) and phosphorus (P) loading and climatic warming can promote their proliferation and toxicity (Van de Waal et al., 2009; Gobler et al., 2016; Visser et al., 2016; Burford et al., 2019). There is also evidence that biotic interactions with heterotrophic bacteria can influence cyanoHAB dynamics (Shen et al., 2011; Bagatini et al., 2014; Van Wichelen et al., 2016).

The microbial consortia associated with algal blooms consist of compositionally and functionally distinct assemblages of free-living bacterioplankton and particle-attached bacteria (Mayali and Azam, 2004). *Microcystis* naturally occurs in large colonies (often > 100 μm) held together by a polysaccharide mucilage (Reynolds et al., 1981; Amemiya et al., 1988; Worm and Søndergaard, 1998) known to harbor diverse epiphytic and embedded bacteria (Kuentzel, 1969; Hoppe, 1981; Worm and Søndergaard, 1998; Parveen et al., 2013). Such colonies may represent a favorable microenvironment for co-occurring bacteria, providing protection from predation and phage infection (Casamatta and Wickstrom, 2000; Dziallas and Grossart, 2012) as well as organic carbon, nutrients, and oxygen (Paerl, 1984; Jiang et al., 2007; Briand et al., 2016). In turn, bacteria may supply algal cells with growth factors (i.e., vitamin B12; Gibson and Smith, 1982; Croft et al., 2005; Li et al., 2018), CO_2_, and regenerated nutrients (Lange, 1967; Harke et al., 2016) and may aid in organic matter decomposition (Harke et al., 2016) and removal of toxic metals (Caldwell, 1979). Shen et al. (2011) suggested that select bacteria may be involved in inducing colony formation. Numerous generalist and host-specific pathogenic (Yoshida et al., 2008; Coloma et al., 2017) and lytic bacteria (Van Wichelen et al., 2016) and references within) such as *Bdellovibrio* have also been identified within *Microcystis* colonies. Algicidal bacteria along with bacteria capable of degrading complex organic molecules have been associated with *Microcystis* bloom decline (Van Hannen et al., 1999; Shao et al., 2014; Berg et al., 2018), among which microcystin-degrading bacteria (Bourne et al., 1996; Park et al., 2001; Maruyama et al., 2003) have been a focus of research as they may influence bloom toxicity (Ho et al., 2007). Due to the complex nature of these associations, however, the precise nature of bacterioplankton within *Microcystis* colonies are not fully understood.

Since bacterial composition strongly shapes the functional potential of microbiomes there has been a great interest in understanding the compositional diversity and dynamics of bacterial assemblages during *Microcystis* blooms. Earlier studies used PCR/DGGE (Riemann and Winding, 2001; Shi et al., 2009, 2012; Shen et al., 2011; Dziallas and Grossart, 2012; Parveen et al., 2013), T-RFLP (Eiler and Bertilsson, 2004; Li et al., 2011) and FISH (Maruyama et al., 2003; Dziallas and Grossart, 2012) to assess *Microcystis* colony-associated microbiomes. In recent years, several studies have been using high-throughput sequencing approaches including next generation amplicon sequencing (Wilhelm et al., 2011; Cai et al., 2014; Louati et al., 2015), metagenomics (Li et al., 2018) and metatranscriptomics (Berg et al., 2018), providing a more comprehensive and high resolution understanding of bacterial communities. Such studies have identified distinct microbial assemblages associated with different cyanobacteria genera (Parveen et al., 2013), species and strains present (Maruyama et al., 2003; Shi et al., 2009; Louati et al., 2015; Li et al., 2018), as well as community variations during different stages of blooms (Van Hannen et al., 1999; Riemann et al., 2000; Parveen et al., 2013; Berg et al., 2018). Differences have been observed between the *Microcystis* colony co-occurring and free-living consortia, with members of the α-, β-, and γ-*proteobacteria*, and *Bacteroidetes* often enriched within the colonies (Cai et al., 2014; Shao et al., 2014; Akins et al., 2018). However, to date few studies have investigated compositional differences of free-living and colony-associated bacteria in natural systems (Kapustina, 2006; Shi et al., 2012; Parveen et al., 2013), and those that have were often over limited spatial and/or temporal scales (Yang et al., 2017; Akins et al., 2018). Further, while there has been extensive research on the impacts of nutrient loading and climatic warming on cyanobacteria composition and proliferation, there remains a limited understanding of how these parameters shape the compositions of naturally occurring *Microcystis*-associated bacteria communities and their functional potential (Wilhelm et al., 2011; Dziallas and Grossart, 2012). Such comparisons could provide important insight on selection and interactions of colony-associated bacteria over different stages of bloom cycle or environmental conditions and their potential roles in bloom dynamics.

Here, we used next generation amplicon-sequencing targeting the 16S rRNA gene to assess the bacteria assemblages associated with *Microcystis* blooms across two temperate North American lakes, with one site sampled for 8 months over a >25°C temperature range. Using a size-fractionation and separation technique, we examined free living (<20 μm; FL), *Microcystis* colony-associated (physically isolated, >20 μm; MCA), and whole (W) bacteria assemblages aiming to: (1) Identify differences in the composition and diversity of these microbiomes between fractions, across lakes, and over time, (2) Identify spatial-temporal variations in these communities related to environmental conditions (i.e., temperature, N and P availability), and (3) Investigate potential functional groups associated with the *Microcystis* colonies focusing on groups that could alter their survival under adverse conditions (i.e., diazotrophs) and impact bloom toxicity (i.e., microcystin-degrading bacteria).

## Materials and Methods

### Study Sites and Field Surveys

**FIGURE 1:**
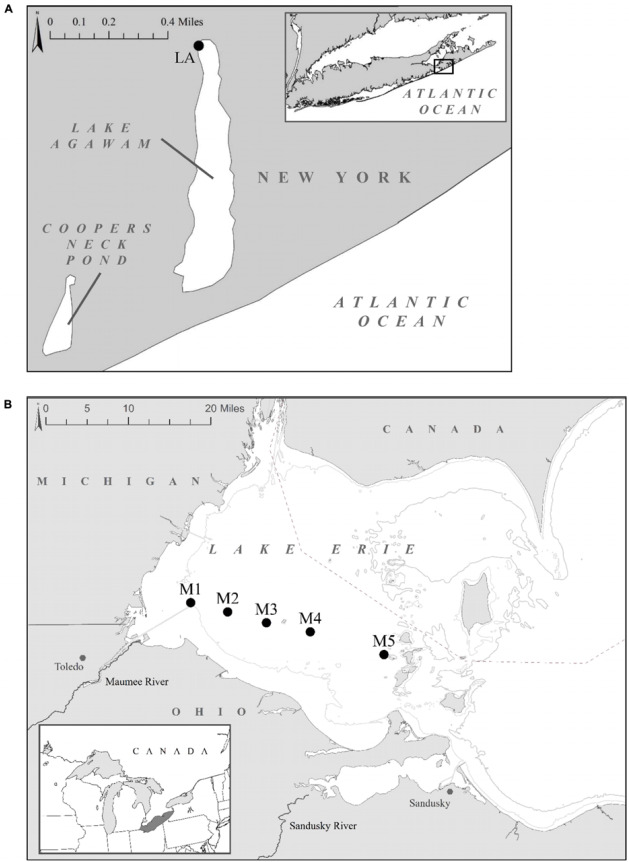
Sampling locations: **(A)** Lake Agawam time series study site. **(B)** Lake Erie study sites located across the Maumee Bay region (M1–M5) in the western basin. The right insert displays Lake Erie’s location within the great lakes. NASA’s MODIS- Aqua data cyanobacterial Index composite images of the bloom extent and intensity on the sampling dates are available at https://www.glerl.noaa.gov/res/HABs_and_Hypoxia/lakeErieHABArchive/. GPS coordinates for sampling sites are listed in Supplementary Table 1.

To examine the dynamics of *Microcystis*-associated microbiomes, prokaryotic communities were investigated during blooms in two North American temperate lakes, Lake Agawam and Lake Erie, in 2017. Lake Agawam (LA) is a small, shallow, eutrophic lake located on Long Island, NY (United States) and is prone to dense, annual *Microcystis*-dominated cyanobacteria blooms that often begin in the spring, as the lake warms with blooms sometimes persisting for more than 8 months (Gobler et al., 2007; Davis et al., 2009). Lake Agawam was monitored weekly from May 2017 through January 2018 at a site on the northern end of the lake (LA; 40.88148, −72.39274; Figure 1A). Lake Erie (LE; United States) is the smallest [by volume; (Davis et al., 2012), warmest (Stumpf et al., 2012), and most eutrophic (Mortimer, 1987; Steffen et al., 2014)] of the Laurentian Great Lake and has experienced intensifying *Microcystis*-dominated blooms since the 1990s (Michalak et al., 2013; Steffen et al., 2014; Ho et al., 2017). Extensive annual *Microcystis* blooms typically occur in LE’s western basin in late summer through early fall (Stumpf et al., 2012), fueled by non-point nutrient loading from the Maumee River (Michalak et al., 2013; Kane et al., 2014). In 2017, a severe *Microcystis* bloom developed in the western Basin in late July and persisted through late October, peaking in August and again in mid-September (National Oceanic and Atmospheric Administration, 2017a). A spatial survey of the bloom was conducted aboard the *R/V* Erie Monitor (The Ohio State University) on September 18 and resampled on September 21 at five sites (Supplementary Table 1) ranging from the Maumee river (M1) to the Bass Islands (M5; Figure 1B). Sites were selected to capture a range of cyanobacteria densities approximated from the September 18 and 21, 2017 MODIS satellite images (National Oceanic and Atmospheric Administration, 2017b, c) and confirmed on-site with a BBE Moldenke Fluoroprobe (*see below*; Beutler et al., 2002; Chaffin et al., 2013; Harke et al., 2015).

On each date/site sampled, surface water temperature was measured using a handheld YSI sonde (model 556) and 1 L of subsurface water (∼0.25 m) was collected for nutrient and microbial community analysis. Duplicate samples for analysis of total (whole water) and dissolved (through a combusted EMD Millipore APFB glass fiber filter) nutrients and total microcystin (whole water) were collected and stored at −20°C until further processing (see *section “sample analysis” for more details*). Phytoplankton communities were fluorometrically assessed with a BBE Moldenke Fluoroprobe to estimate cyanobacterial abundance in terms of chlorophyll *a* (Chl *a*) based on differential fluorescence of photosynthetic accessory pigments among cyanobacteria, green algae, brown algae (e.g., diatoms, dinoflagellates, raphidophytes, and haptophytes) and cryptophytes (Beutler et al., 2002; Chaffin et al., 2013; Harke et al., 2015). The signal of each channel was affirmed with >50 cultures of diatoms, cyanobacteria, dinoflagellates, raphidophytes, green algae, and haptophytes (Jankowiak et al., 2019). Due to significant cross over of the cyanobacteria signal into the cryptophyte channel, cryptophyte levels were not included in this study.

### Heterotrophic Bacteria Fractionation

To compare the *Microcystis* colony-associated microbiomes to other particle-attached and free-living bacteria, bloom water was fractionated into three size fractions targeting the colony-attached (MCA), free-living (FL), and whole (W) bacteria assemblages using an approach modified from Kapustina (2006) and Cai et al. (2013). Specifically, *Microcystis* colonies were isolated by filtering 1 – 4 L of water, dependent on cyanobacterial densities, onto a 20-μm nylon mesh sieve, based on the operational definition provided by Worm and Søndergaard (1998) while allowing free-living bacteria to pass into the filtrate. To purify the *Microcystis* colonies from other large particles collected on the filter, the biomass was rinsed with, and resuspended in, 200 ml of bacteria-free, 0.2-μm filtered lake water to allow any large, non-*Microcystis* particles to settle out of suspension and *Microcystis* colonies to float to the surface due to their high buoyancy. The colonies were then skimmed off the surface using a 50 mL serological pipet and resuspended twice more, as described to further purify the colony fraction. An aliquot of this fraction was then preserved with Lugol’s iodine solution (5% v/v) and examined via microscopy to confirm there was no contamination of other large particles or phytoplankton. Free-living bacteria were isolated from the 20-μm filtrate while well-mixed whole water was collected to capture all bacteria. Triplicate samples from each fraction (FL: 50 mL, MCA: 50 ml, W: 20–100 mL) were filtered onto 0.22 μm polycarbonate filters and immediately stored at −80°C until DNA extraction (*see section “DNA Isolation, Sequencing, and Analysis” below*). Additionally, samples from the W and FL fractions were preserved with Lugol’s iodine solution (5% v/v) for microscopic examination to confirm that *Microcystis* was the dominate cyanobacteria in the whole fraction and that there was no *Microcystis* contamination in the FL fraction.

### Sample Analysis

Nutrient samples were analyzed for nitrate, ammonium, orthophosphate, total nitrogen (TN) and total phosphorus (TP) on a Lachat Instruments autosampler (ASX-520 series) using standard wet chemistry (Valderrama, 1981; Jones, 1984; Parsons, 2013). Recovery of standard reference material of at least 95 ± 10% was achieved for all nutrient analyses. Total microcystins were extracted from whole water samples by chemical lysis using the Abraxis Quicklyse Cell Lysis Kit, then quantified using the congener independent (Fischer et al., 2001) Abraxis Microcystin/Nodularian (ADDA) ELISA (enzyme-linked immunosorbent assay) kit per the manufacturer’s instructions. Concentrations were measured on a SpectraMax plus 384 plate reading spectrophotometer, calculated using a logarithmic curve, and reported in microcystin LR-equivalents. This assay provided a 98.6 ± 5% average recovery of samples spiked with known concentrations of microcystin-LR standard from the National Research Council of Canada.

### DNA Isolation, Sequencing, and Analysis

For molecular analyses, total nucleic acids were extracted from the field samples using the DNeasy PowerWater Kit (Qiagen; Venlo, Netherlands) per the manufacturer’s instructions. The extracted nucleic acids were re-suspended in 100 μl of sterile PCR water and the quality and quantity of the double-stranded DNA was assessed for using a Nanodrop spectrophotometer and Qubit^®^ fluorometer with a dsDNA BR Assay kit, respectively, per the manufacturer’s instructions. Samples were normalized to the lowest concentration of DNA among samples and stored at −80°C until PCR amplification and amplicon sequencing. Sequencing was performed at Molecular Research Laboratories (Shallowater, TX, United States) following the method described in Jankowiak et al. (2019) on an Illumina MiSeq (2 × 300 bp). To examine the prokaryotic assemblages, the V4 region of the 16S SSU rRNA gene (∼390 bp) was amplified using the universal primer set 515F: 5′-GTGYCAGCMGCCGCGGTAA-3′ (Parada et al., 2016) and 806R: 5′-GGACTACNVGGGTWTCTAAT-3′ (Apprill et al., 2015).

The 16S sequence data was processed using the Quantitative Insights Into Microbial Ecology QIIME 1 (v1.9.1) and QIIME 2 (v2018.6.0) microbiome analysis software packages following the “Moving Pictures” pipeline (Caporaso et al., 2010; Bolyen et al., 2018). Briefly, the raw FASTA and quality files of the joined paired-end reads were merged to create a FASTQ file using Phred33 conversion software (v17.03.22; Phred 33 [Computer software], 2017) and the reads were trimmed of their sample identification barcodes in QIIME 1. The reads were then de-multiplexing into their respective samples based on the associated barcodes in QIIME 2 using the DEMUX plugin and depleted of both primers using the Cutadapt plugin (Martin, 2011). The resulting split library output was filtered for chimeric sequences, denoised, and dereplicated into 100% amplicon sequence variants (ASVs) using DADA2 (Callahan et al., 2016). While several prior studies have used amplicon sequencing to describe *Microcystis*-associated microbiomes (Cai et al., 2014; Louati et al., 2015; Akins et al., 2018), this is, to the best of our knowledge, the first to use this high level of stringency regarding identification of microbes associated with *Microcystis* blooms. The ASV representative sequences were assigned taxonomies using a trained classifier and the q2-feature-classifier (Bokulich et al., 2018) and classify-sklearn (Pedregosa et al., 2011) plugins. To create the classifier a Naive Bayes classifier was trained on the SILVA rRNA (16S SSU) release v132 reference database (Quast et al., 2012) using the 99% 16S only rep set FASTA and majority consensus seven-level taxonomy files trimmed to the primer region with high recall settings (0.5 confidence level, 11 k-mer length). To confirm taxonomic assignment, representative sequences of the most abundant ASVs were identified using NCBI BLAST (Altschul et al., 1990). Post-processing, the 16S dataset was refined into exclude all mitochondria and chloroplast annotated features and the resulting dataset was split into cyanobacterial and non-cyanobacterial reads to separately analyze cyanobacterial and bacterial communities. Sequence data from this study has been deposited to NCBI SRA database (SRA bioproject PRJNA601166, Accession: SRX7554361-SRX7554236)^1^.

### Statistical Analyses

All statistical analyses were performed in QIIME 2 v2018.6 (Bolyen et al., 2018) unless otherwise noted. ASV inferred alpha (observed ASVs, Shannon richness and Pielous evenness) and beta (Bray–Curtis dissimilarity) diversity metrics were calculated for the bacteria and cyanobacteria datasets rarefied to a sampling depth of 20,633 reads using the QIIME 2 core metrics pipeline. This sampling depth was chosen as it was the number of reads in the sample with the lowest read count. Significant differences in the alpha diversity metrics among lakes and size fractions were assessed using Kruskal–Wallis pairwise tests with multiple comparison correction (Kruskal and Wallis, 1952) and significant correlations with the physio-chemical parameters (surface temperature, total and dissolved nutrients, Fluoroprobe derived cyanobacteria abundance, and microcystin concentrations) were assessed using Spearman correlations in R v3.2.3 (R Core Team, 2013).

Multivariate statistical approaches were employed to analyze beta diversity metrics and identify differences among the bacterial and cyanobacterial community structures (composition/abundance) between samples. Specifically, principal coordinates analysis (PCoA) was conducted on the ASV inferred Bray-Curtis dissimilarities to identify groups of samples with similar community compositions. Significant differences in community composition and community homogeneity between lakes and size fractions were assessed using PERMANOVA (Permutational multivariate analysis of variance) and PERMDISP (Permutational multivariate analysis of dispersion), respectively, with 999 permutations calculated per test. SIMPER (Similarity Percentage) analysis was then performed in PRIMER v7 (Clarke and Gorley, 2015) for groups found to be significantly different to identify the average community composition dissimilarity between them. Additionally, significant correlations of the community dissimilarities with the physio-chemical parameters and biological responses (surface temperature, total and dissolved nutrients, Fluoroprobe-derived cyanobacteria abundance, microcystin concentrations and growth rate) were assessed using Mantel tests (999 permutations) and Spearman correlations.

Differential abundance analysis was used to identify significant differences in the abundances of the bacterial ASVs between the size fractions within each lake. Significant log fold changes in abundances of individual ASVs were assessed using the Phyloseq and DESeq packages (Anders and Huber, 2010; McMurdie and Holmes, 2013, 2014) in R. Briefly, raw 16S read abundances were normalized with the median ration method then modeled using a negative binomial distribution with parametric fitting of the dispersions. Significant log_2_ fold changes in abundance (α = 0.05) were determined with Wald significance testing and P-values were adjusted using the Benjamini–Hochberg procedure to correct for multiple testing. This was complemented by analysis with Venny software v2.1 (Oliveros, 2007) to identify ASVs shared across lakes within each fraction and ASVs shared among the size fractions within each lake as well as to determine the core (included in 100% of the group samples) and unique (found only in that group) features of each group (Size: W, MCA, FL; Lake: LA, LE). Gneiss analysis (Morton et al., 2017) was used to assess differentially abundant groups of covarying ASVs over time and space among the size fractions. Unlike other differential abundance analyses, gneiss analysis uses balances (log ratios of subcommunities) instead of relative abundances, in which a change in one taxon influences the abundance of others, providing a more accurate compositional interpretation (Morton et al., 2017). Specifically, a pseudocount (+1) was added to the abundances prior to ASVs clustering based on co-occurrence using Ward hierarchical clustering and then the log ratios between the resulting clusters was calculated using isometric log ratio (ILR) transformation to obtain balances. A multivariate response linear regression was then performed on the balances, examining the effects of temperature, cyanobacteria abundance, nitrogen (NO_3_^–^, NH_4_^+^, TN) and phosphorus (PO_4_^3–^, TP) concentrations to identify potential environmental drivers of the subcommunities.

To investigate the potential functional capabilities among the heterotrophic bacteria communities, PICRUST (Phylogenetic Investigation of Communities by Reconstruction of Unobserved States) software (Langille et al., 2013; Louca and Doebeli, 2017; Czech and Stamatakis, 2018; Barbera et al., 2018) was used to create predicted composite metagenomes for each sample. PICRUSt uses a phylogenetic reconstruction algorithm with marker gene data, such as 16s rRNA, and the KEGG reference genome database to predict the abundance of gene families within a community. Prior to analysis all cyanobacteria-taxonomically assigned reads were removed and the 16S ASV abundances were normalized (relative abundances). Resulting predicted metagenomes were visualized with PCoA analysis and the differential abundance of KEGG gene families of particular interest (i.e., genes associated with N and P cycling) were further investigated using STAMPS software v2.1.3 (Parks et al., 2014). To further assess the metagenomic predictions made via PICRUSt analyses, the total relative abundance of taxa with known sequences of the nitrogen fixation *nif*H gene in the NCBI nucleotide database were manually compared among the fractions. Specifically, all *nifH* sequences classified as heterotrophic bacteria in the NCBI nucleotide database and their associated taxonomies were downloaded as a reference database. The database was then used to filter the QIIME ASV frequency table based on taxonomic assignment to extract abundances of all potentially *nif*H containing taxa. A similar approach was used to examine the abundance of genes associated with microcystin degradation that were not annotated in the KEGG database for PICRUSt analysis. Specifically, the *mlr* gene cluster (*mlr*ABCD) which encodes a MC-LR cleavage pathway in bacteria (Bourne et al., 1996, 2001) and putative glutathione S-transferase (GST) genes, which are key genes in xenobiotic metabolism and involved in microcystin degradation (Campos and Vasconcelos, 2010), were used to identify potential microcystin-degrading bacteria in this study.

## Results

### Physiochemical Conditions and Cyanobacteria Bloom Characteristics

**FIGURE 2:**
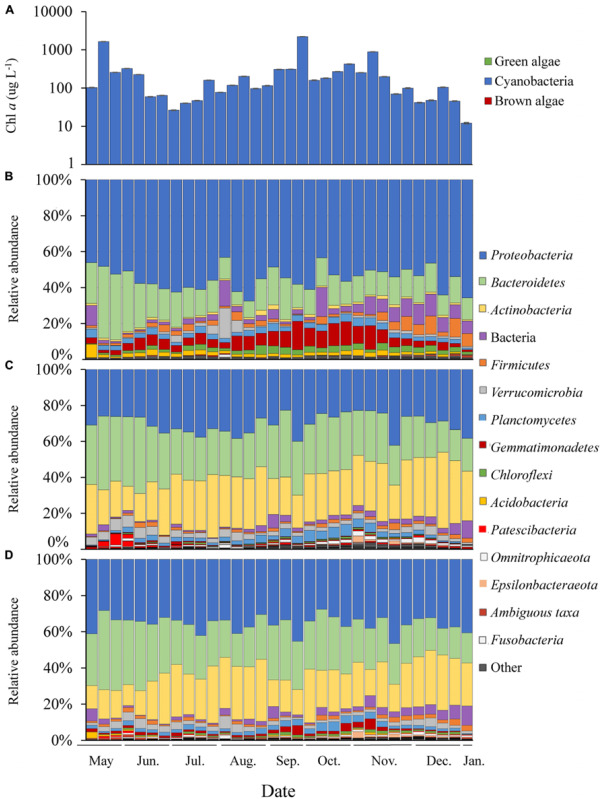
Time series of Lake Agawam cyanobacteria and bacteria abundance data. **(A)** Chl *a* absolute abundance of the cyanobacteria, green and brown algal pigment classes determined via Fluoroprobe shown on a log scale. Bacteria phylum relative abundances determined via 16S sequencing in the **(B)** *Microcystis* colony, **(C)** free-living and **(D)** whole water fractions. All low abundance phyla have been grouped into the “other” category.

In LA, a dense bloom of *Microcystis* was present from May 8, 2017 until January 5, 2018 (Figure 2) when the bloom declined following a significant drop in temperature (Table 1). During this time, cyanobacteria concentrations were above the New York State Department of Environmental Conservation bloom threshold of 25 μg L^–1^ cyanobacterial Chl *a* on all dates, with bloom peaks occurring on May 17th (1,637 μg L^–1^), September 26th (2,209 μg L^–1^) and November 6th (884 μg L^–1^; Figure 2). The early (spring) and extended nature of *Microcystis* blooms in Lake Agawam are common, partly due to its eutrophic, shallow and thus warmer nature (Gobler et al., 2007; Davis et al., 2009). Microcystin concentrations were above the US EPA recreational (4 μg L^–1^) guideline (EPA, 2015, 2016) on over 88% days sampled, peaking at 375 μg L^–1^ on September 26th (Table 1). This bloom persisted through a >25°C seasonal change in temperature (1.4 – 26.6°C) and dynamic nutrient levels during the 8-month survey (Table 1). On average, nitrogen concentrations were lower from May to October (nitrate 2.14 ± 2.86, ammonia 1.71 ± 1.07, TN 150 ± 84.7 μM) and increased into the late fall and winter (nitrate 30.7 ± 19.6, ammonia 32.3 ± 23.1, TN 243 ± 117 μM; Table 1). TP averaged 5.51 ± 4.18 μM throughout the sampling period with occasional spikes tracking blooms peaks while orthophosphate was below the detection limit (0.13 μM) on >80% of the days sampled (Table 1).

**TABLE 1:** *In situ* physiochemical conditions and nutrient concentrations during the Lake Agawam (LA) time series and Lake Erie (LE) transects (sites M1–M5, river to Bass Islands).

**Site**	**Date**	**Toxin (μg L^–1^)**	**Surface Temperature (°C)**	**Nitrate (μM)**	**Ammonia (μM)**	**Phosphorus (μM)**	**TN (mg N L^–1^)**	**TP (μg P L^–1^)**
LA	17-05-2017	317	19.6	10.57 ± 0.3	0.36 ± 0.09	BDL	47.11 ± 1	0.55 ± 0.05
LA	23-05-2017	75.02	18.8	0.12 ± 0.02	0.61 ± 0.2	BDL	221.56 ± 30.9	6.41 ± 0.29
LA	30-05-2017	131.43	18.6	0.87 ± 0.23	NA	BDL	238.91 ± 10	7.37 ± 0.33
LA	06-06-2017	28.3	17.5	6.48 ± 0.26	1.37 ± 0.42	BDL	227.34 ± 8.18	6.59 ± 0.29
LA	13-06-2017	16.15	25	BDL	0.87 ± 0.16	BDL	90.03 ± 0.64	2.72 ± 0.04
LA	19-06-2017	15.5	23.1	0.53 ± 0.08	1.88 ± 0.39	BDL	94.02 ± 1.54	2.72 ± 0.08
LA	26-06-2017	3.57	27.7	0.4 ± 0.01	NA	BDL	63.18 ± 1	2.32 ± 0.17
LA	03-07-2017	6.64	NA	0.5 ± 0.01	1 ± 0.23	BDL	74.93 ± 1.27	2.43 ± 0.02
LA	17-07-2017	11.15	27.7	0.31 ± 0.01	1.99 ± 0.29	BDL	65.94 ± 0	2.94 ± 0.25
LA	25-07-2017	16.59	24.4	0.68 ± 0.2	0.68 ± 0.09	BDL	61.18 ± 4	2.53 ± 0.14
LA	31-07-2017	83.92	26.6	1.73 ± 0.26	3.39 ± 1.13	BDL	93.44 ± 0	3.99 ± 0.24
LA	07-08-2017	37.51	23.9	4.64 ± 1.12	2.89 ± 0.07	BDL	NA	6.38 ± 0.16
LA	15-08-2017	46.21	26	0.9 ± 0.31	1.57 ± 0.53	BDL	211.92 ± 4.54	9.46 ± 0
LA	22-08-2017	28.48	26.3	4.14 ± 0.61	3.3 ± 0.46	BDL	132.89 ± 5.45	5.63 ± 0.16
LA	28-08-2017	28.87	26.2	0.45 ± 0.12	1.35 ± 0.34	1.36 ± 0	NA	3.72 ± 0.12
LA	06-09-2017	82.27	22.4	4.62 ± 0.33	1.67 ± 0.2	0.37 ± 0.07	184.29 ± 19.99	9.09 ± 0.86
LA	11-09-2017	78.8	NA	0.99 ± 0.22	3.07 ± 0.43	BDL	230.56 ± 12.72	8 ± 0.04
LA	26-09-2017	302.86	24.2	0.09 ± 0	3.52 ± 0.23	BDL	336.58 ± 22.72	14.62 ± 1.11
LA	03-10-2017	38	20.9	0.49 ± 0.18	0.73 ± 0.17	BDL	172.73 ± 0	4.68 ± 0.05
LA	11-10-2017	NA	20.2	4.37 ± 0.32	39.61 ± 0.27	BDL	206.78 ± 0.91	6.02 ± 0.12
LA	17-10-2017	42.37	15.9	9.69 ± 0.27	6.3 ± 0.47	0.27 ± 0.06	183.01 ± 1.82	4.94 ± 0.09
LA	24-10-2017	85.07	18.2	0.44 ± 0.02	2.02 ± 0.12	BDL	280.67 ± 0	8.26 ± 0.16
LA	01-11-2017	62.87	13.7	18.37 ± 1.42	15.19 ± 2.33	BDL	246.25 ± 0	4.94 ± 0.01
LA	06-11-2017	171.35	15.2	23.56 ± 0.43	5.81 ± 0.45	BDL	599.37 ± 25.44	21.34 ± 0.9
LA	13-11-2017	374.5	7.8	33.02 ± 0.18	24.38 ± 0.54	1.14 ± 0.07	217.98 ± 7.27	3.97 ± 0.07
LA	22-11-2017	15.74	10.6	NA	NA	NA	NA	NA
LA	27-11-2017	25	7.8	39.72 ± 3.99	28.2 ± 3.89	0.87 ± 0	176.86 ± 0	3.31 ± 0
LA	04-12-2017	10.49	8.2	58.07 ± 0.3	74.72 ± 1.09	BDL	193.56 ± 3.63	1.75 ± 0.03
LA	11-12-2017	6.81	6.2	49.15 ± 0.5	49.42 ± 0.94	BDL	165.29 ± 0	2.25 ± 0.1
LA	19-12-2017	9.99	3.1	51.63 ± 0.53	48.89 ± 0.77	BDL	221.19 ± 10	3.98 ± 0.05
LA	26-12-2017	12.27	3.2	49.74 ± 0.37	60.61 ± 2.61	BDL	179.43 ± 3.63	2.52 ± 0.08
LA	05-01-2018	NA	1.4	NA	NA	NA	NA	NA
LE M1	18-09-2017	5.49	20.8	0.46 ± 0.14	3.28 ± 0.47	BDL	34.98 ± 3.09	1.16 ± 0.11
LE M2	18-09-2017	0.46	20.6	3.7 ± 0.24	3.44 ± 1.11	BDL	327.85 ± 15.45	8.41 ± 0.45
LE M3	18-09-2017	3.79	20.5	0.15 ± 0	0.99 ± 0.03	BDL	212.84 ± 0	5.88 ± 0
LE M4	18-09-2017	2.69	20.5	1.7 ± 0.24	1.42 ± 0.13	BDL	60.43 ± 7.09	1.79 ± 0.12
LE M5	18-09-2017	2.83	20.7	5.72 ± 0.23	0.34 ± 0.04	BDL	53.43 ± 4.09	1.2 ± 0.15
LE M1	21-09-2017	2.72	21.2	0.57 ± 0.07	0.81 ± 0.2	BDL	NA	NA
LE M2	21-09-2017	0.27	20.9	15.21 ± 0.38	0.6 ± 0.13	BDL	NA	NA
LE M3	21-09-2017	0.55	20.8	5.78 ± 0.23	0.12 ± 0	BDL	NA	NA
LE M4	21-09-2017	4.52	20.8	0.75 ± 0.08	BDL	BDL	NA	NA
LE M5	21-09-2017	2.43	21.1	3.52 ± 0.27	0.15 ± 0.02	BDL	NA	NA

*Values are displayed as mean ± SD.*

**FIGURE 3:**
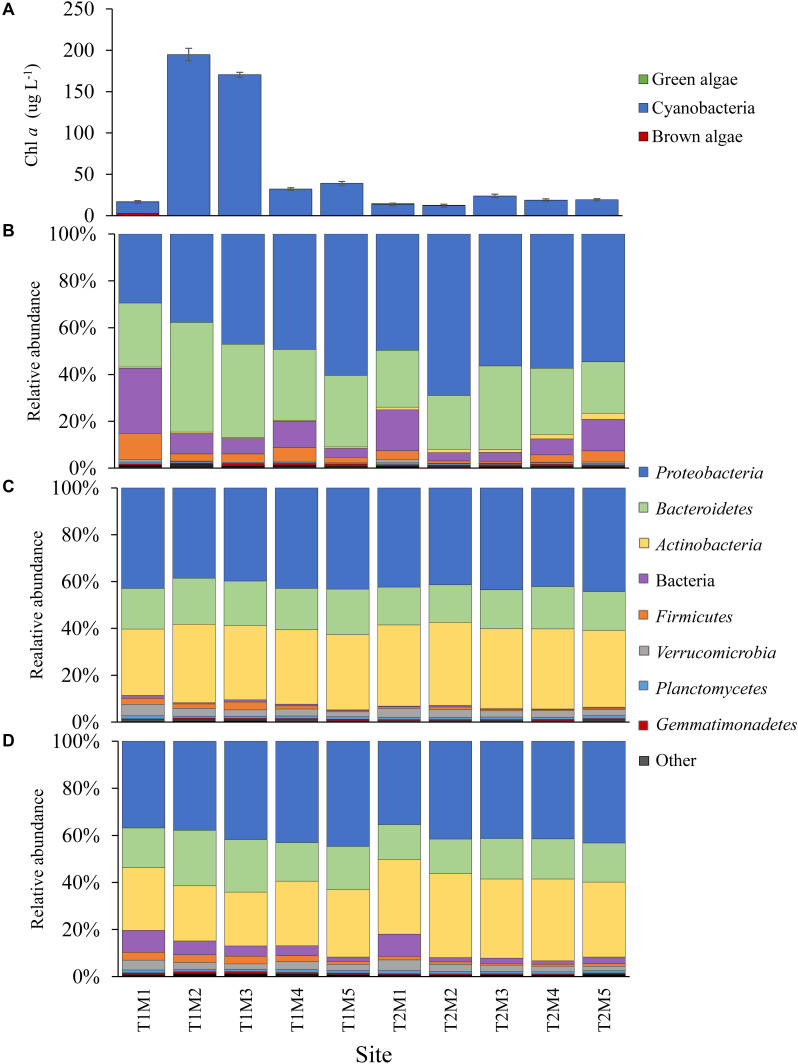
Cyanobacteria and bacteria abundance data from the Lake Erie spatial surveys. **(A)** Chl *a* absolute abundance of the cyanobacteria, green and brown algal pigment classes determined via Fluoroprobe. Bacteria phylum relative abundances determined via 16S sequencing in the **(B)** *Microcystis* colony, **(C)** free-living and **(D)** whole water fractions. In the Site abbreviation T denotes the transect (T1-September 18th, T2-September 21st) and M denotes the sampling site. All low abundance phyla have been grouped into the “other” category.

In LE, on September 18th, cyanobacteria concentrations peaked near the Maumee River (site M2 = 195 ± 7.59 μg L^–1^) and declined to the east, while on September 21st cyanobacteria concentrations were higher offshore (peak at M3, 23.9 ± 2.09 μg L^–1^) but were lower than concentrations observed during first transect (Figure 3). Surface water temperature was consistent across sites and dates (20.79 ± 0.23°C) and microcystin concentrations ranged from 0.27 to 5.49 μg L^–1^ (Table 1). Along both transects ammonium concentrations generally decreased with distance from the Maumee River and were higher during the first transect (1.89 ± 1.39 and 0.42 ± 0.34 μM) while nitrate concentrations varied across sites and were lower during the first transect (2.35 ± 2.35 and 5.17 ± 6.01 μM; Table 1). TN and TP concentrations paralleled cyanobacteria levels, ranging from 35.0 to 328 μM and 1.16 to 8.41 μM, respectively (Table 1). Orthophosphate concentrations were below the detection limit (0.13 μM) in all samples (Table 1). In both lakes, differential fluorescence analyses using the BBE Fluoroprobe indicated cyanobacteria made up >90% of the phytoplankton community in all samples (Figures 2, 3) and microscopic analyses indicated all the cyanobacteria communities were dominated by *Microcystis* (by volume). Cyanobacterial biomass was strongly and significantly correlated with TN and TP concentrations (*p* < 0.001, ρ = 0.63, 0.71) in LA and significantly inversely correlated with surface temperature in LE (*p* < 0.001, ρ = −0.8; Supplementary Table 2).

### Microbiome Sequencing

The 96 LA and 30 LE samples collectively generated a total of 7,922,628 sequences after joining and quality filtering with an average length of 368 bp. The sequences clustered into 7,447 ASVs with 100% sequence identity, among which there were 420 mitochondrial/chloroplast ASVs that were not considered for further analysis, 6,923 bacterial ASVs, and 104 cyanobacterial ASVs. Among lakes, 5,967 bacterial and 73 cyanobacterial ASVs were identified in the LA communities while 1,496 bacterial and 46 cyanobacterial ASVs were present in the LE communities.

### Cyanobacteria Assemblages Within Fractions

**FIGURE 4:**
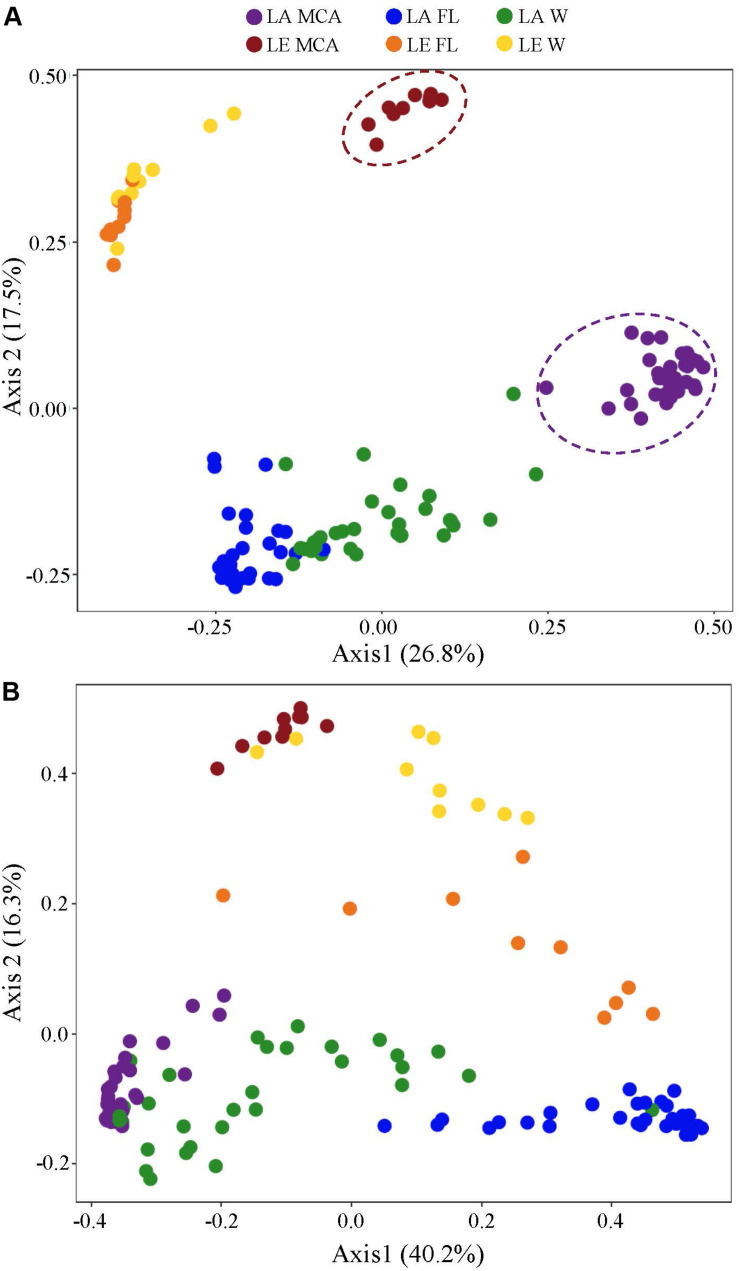
Principal coordinates analysis (PCoA) conducted on ASV abundances showing the dissimilarity of the **(A)** heterotrophic bacteria and **(B)** cyanobacteria community compositions among all Lake Agawam time series and Lake Erie spatial survey samples. Colors denote the Lake (LA or LE) and size fraction (MCA, FL, W) of the sample. Circles indicate significant clustering of the MCA communities.

Cyanobacterial communities determined via 16S rRNA sequencing were significantly different between size fractions in both lakes (*p* < 0.001; Supplementary Table 3), with the MCA and FL fractions having the most dissimilar compositions (LA: 35.01%, LE: 47.44% dissimilarity) and the MCA and W compositions being highly similar (LA: 22.2%, LE: 21.9% dissimilarity; Figure 4 and Supplementary Table 4). *Microcystis* (identified as *Microcystis* PCC-7914) was a dominant component of cyanobacterial communities in all samples (Supplementary Figures 1, 2), and was significantly more abundant (*p* < 0.001, Supplementary Figure 3) in the MCA and W fractions accounting for nearly a quarter of the cyanobacteria reads in LA (Supplementary Figure 1) and half in LE (Supplementary Figure 2), compared to only 7.0% (Supplementary Figure 1) and 35% (Supplementary Figure 2) in the FL fraction in LA and LE, respectively. The small filamentous *Pseudanabaena* was predominantly the most abundant cyanobacteria in all fractions across lakes, on average accounting for 65–75% and 32–47% of the cyanobacteria reads in LA and LE, respectively (Supplementary Figures 1, 2). Together, these two genera accounted for over 70% of the cyanobacterial communities in all samples (Supplementary Figures 1, 2). The pico-cyanobacterium *Cyanobium* was found nearly exclusively in the FL communities (*p* < 0.001, Supplementary Figure 3), present in high abundances in LA from July 3 - September 6, accounting for 16 – 56% of the cyanobacteria reads (Supplementary Figure 1) and 8.7% of the communities in LE (Supplementary Figure 2), while contributing to less than 1% of the MCA communities. Concurrent with the *Cyanobium* peak in LA, *Dolichospermum* was found in high abundances in the MCA and W fractions from Jun 26 August 22, accounting for 20% and 13% of the cyanobacteria reads, respectively (Supplementary Figure 1), but accounting for <10% of cyanobacteria reads in the LE fractions (W: 7.1%, MCA: 7.5%, FL: 6.8%; Supplementary Figure 2).

The LA cyanobacterial communities were, on average, less diverse than the LE communities, with significantly lower richness and evenness indices in all three fractions (Supplementary Table 5 and Supplementary Figure 4). Within both lakes, the FL cyanobacteria assemblages were the most diverse of the three size fractions, with significantly higher community richness and evenness (Supplementary Table 5 and Supplementary Figure 4) and significantly greater compositional dispersion across samples than the W and MCA communities (Supplementary Table 3 and Supplementary Figure 5). Across lakes, cyanobacteria community diversity (ASVs and Shannon richness) was significantly (*p* < 0.05; ρ = 0.32–0.60) correlated with temperature in all size fractions, but not nutrients (Supplementary Table 6). Further, the diversity of the MCA and FL cyanobacteria communities were significantly correlated with cyanobacteria abundance in LA but were inversely correlated to cyanobacteria abundance in LE (*p* < 0.05; Supplementary Table 6).

### Heterotrophic Bacteria Compositional Differences Among Size Fractions

**FIGURE 5:**
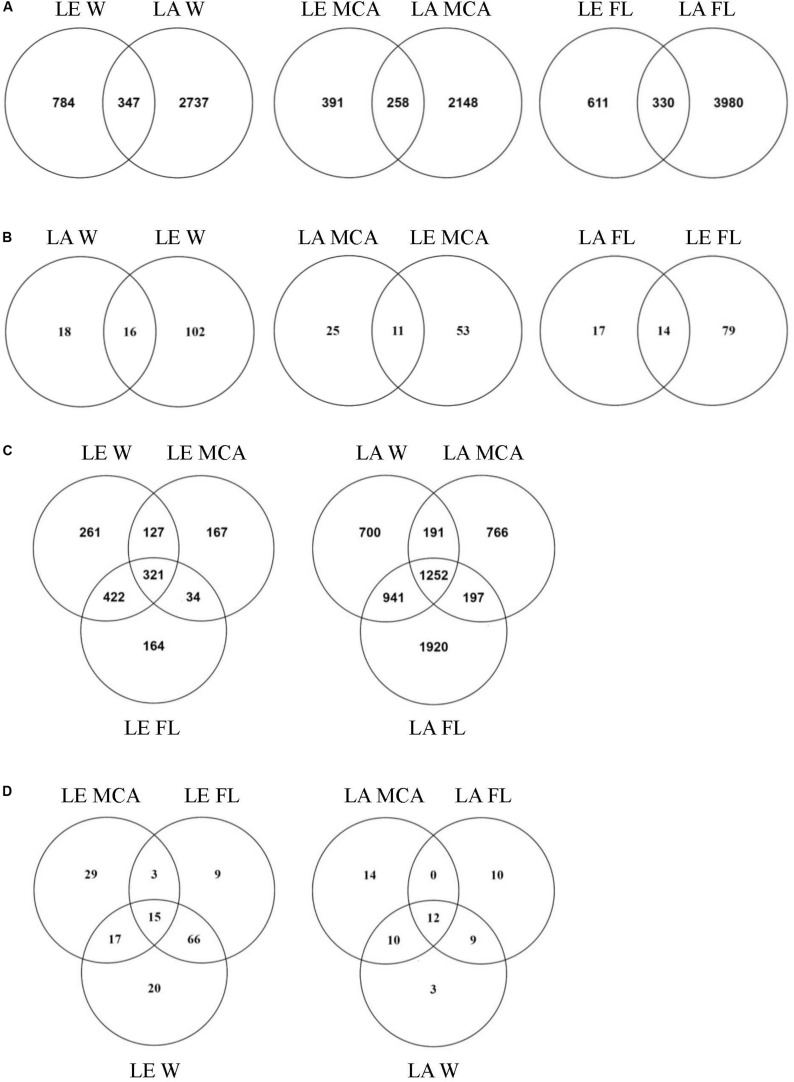
Venn diagrams showing **(A)** the total number of ASVs and **(B)** the number of ASVs in the core microbiome (i.e., ASV found in all samples within the group) shared between Lake Agawam and Lake Erie heterotrophic bacteria communities in the Microcystis colony, free-living and whole water fractions. **(C)** The total number of ASVs and **(D)** the number of ASVs in the core microbiome (i.e., ASV found in all samples within the group) shared between the heterotrophic bacteria communities in the Microcystis colony, free-living, and whole water fractions within Lake Erie and Lake Agawam.

Significantly different heterotrophic bacteria communities were identified in each lake and across all three size fractions within each lake (*p* < 0.001; Supplementary Table 3 and Figure 4). Overall, size fractions explained the largest amount of compositional variation (30%; axis 1, Figure 4). In contrast to the cyanobacteria communities, in both lakes, the W and FL heterotrophic bacterial microbiomes exhibited the most similar compositions (LA: 63.83%, LE: 30.5% dissimilarity) while the MCA communities were the most dissimilar (LA: >75%, LE: >80% dissimilarity to W and FL; Supplementary Table 4), with the strongest differences observed between the MCA and FL microbiomes (f statistic; LA: 46.4%, LE: 59.3%; Supplementary Table 3 and Figure 4). Additionally, the MCA communities were most dissimilar between lakes (f stat = 35.5, Supplementary Table 3) with an average compositional dissimilarity of 88.9% (Supplementary Table 4) and the lowest number of shared ASVs (258 compared to 347 and 330 among the W and FL fractions respectively; Figure 5). However, each fraction was more similar to the same fraction than other size fractions across lakes (Figure 4).

#### Heterotrophic Bacteria in Lake Agawam

Of the 5,967 ASV identified in LA, 1,252 ASVs were shared across size fractions (Figure 5) and 335 were found to differ significantly in abundance between the size fractions. The MCA and FL fractions were the most dissimilar with 312 significantly differentially abundant ASVs (*p* < 0.05), of which 187 and 144 were enriched in the MCA and FL fractions, respectively. Only 36, 31, and 34 ASVs were found in every MCA, FL and W fraction sample (Figure 5) and of those core ASVs, 12 were shared between all fractions while 14, 10 and 3 were unique to the MCA, FL and W fractions, respectively (Figure 5). Of the three fractions, MCA microbiomes were the least diverse, having a significantly lower Shannon richness and fewer ASVs than both the FL and W communities (Supplementary Table 5 and Supplementary Figure 4).

Of the 51 phyla identified in the LA communities, 28 phyla were significantly differentially abundant between the MCA and FL/W factions (Supplementary Figure 6). Among the greatest differences between the MCA and the FL and W microbiomes in LA was due to differences in the abundances of the *Gemmatimonadetes* and *Actinobacteria* phyla. The *Gemmatimonadetes* phylum was significantly enriched in the MCA fraction, being nearly 4.0 log_2_ fold more abundant compared to the other fractions (*p* < 0.001; Supplementary Figure 6) and accounting for up to 16% of MCA communities compare to 0.09–5.9% in the FL and W microbiomes (Figure 2). Conversely, *Actinobacteria* were significantly (5.0 log_2_ fold) depleted in the MCA fraction (*p* < 0.001; Supplementary Figure 6), accounting for nearly a quarter of the FL and W communities while being nearly absent from the MCA fraction (<1%; Figure 2). The *Gemmatimonadetes* consisted primarily of uncultured members of the *Gemmatimonadaceae* family and the *Gemmatimonas* genus while the *Actinobacteria* consisted primarily of the *Frankiales* (*Sporichthyaceae*; FL: 21.2, W: 17.6% of total) and *Micrococcales* (*Microbacteriaceae*; FL: 4.2, W: 4.1% of total) orders.

The *Proteobacteria* and *Bacteroidetes* were the dominant phyla in all samples (Figure 2), but differentially abundant between the fractions. *Proteobacteria* were significantly enriched in the MCA fraction (*p* < 0.001; Supplementary Figure 6) accounting for, on average, 55% of the bacterial reads compared to ∼30% in the FL and W fractions (Figure 2), being comprised primarily of the γ-*proteobacteria* (> 65%), followed by the α- (21.1%) and δ-*proteobacteria* (<10%) in all fractions. The γ-*proteobacteria* were significantly enriched in the MCA fraction (*p* < 0.001; Supplementary Table 7), consisting primarily of the *Burkholderiaceae* (MCA: 43.5% of γ), *Methylophilaceae* (24.6% of γ), and *Nitrosomonadaceae* (19.3% of γ) families. The *Rhizobiales* order was the prominent member of the α*-proteobacteria* in all three fractions but was significantly enriched in the MCA fraction (*p* < 0.05; Supplementary Table 7). On average, the *Bacteroidetes* phylum was significantly depleted in the MCA fraction (*p* < 0.001; MCA: 12.7%, FL: 28%, W: 26.3%; Figure 2 and Supplementary Figure 6) and consisted primarily of the *Cytophagales* order while the *Sphingobacteriales* and *Chitinophagales* were the dominant orders in the FL and W fractions (Supplementary Table 7). An additional 24 phyla were significantly differentially abundant between the MCA and FL/W factions but present at lower abundances (<2% of the reads; Supplementary Figure 6).

### Heterotrophic Bacteria of Lake Erie

At the ASV level, the MCA and FL bacterial microbiomes in LE were the most dissimilar, with 362 significantly differentially abundant ASVs. There were also a large number of significantly different ASVs between the MCA and W (280) while the W and FL fractions were highly similar with only 28 differentially abundant ASVs. Of the 362 significant variants between MCA and FL fractions, 310 were significantly depleted in the MCA fraction while only 61 were significantly enriched in the MCA fraction, primarily belonging to the α- and γ-*proteobacteria*. Of the 1,496 ASVs identified in the LE communities, 321 ASVs were shared across the size fractions, of which 15 ASVs were part of the W, FL and MCA core microbiomes (Figure 5). Individually, the MCA fraction had the smallest core microbiome across samples (64 compared to 93 in FL and 118 in W) but the largest number of unique ASVs (29 compared to 9 in FL and 20 in W; Figure 5). Like the LA communities, the LE MCA communities were the least diverse of the three fractions, with a significantly lower number of ASVs on average, while the FL communities were the least even (Supplementary Table 5 and Supplementary Figure 4).

Among the 49 phyla identified within the LE communities, 10 were significantly differentially abundant within the MCA fraction (*p* < 0.05; Supplementary Figure 6). As was the case for LA, the *Actinobacteria* phylum accounted for the greatest difference between the MCA and the FL microbiomes in LE (∼5 log_2_ fold, Supplementary Figure 6), but the *Gemmatimonadetes* phylum was nearly absent in this system accounting for less than 1% of the communities in all samples (Figure 3). The *Actinobacteria* were, again, significantly depleted (*p* < 0.001; Supplementary Figure 6) in the MCA communities, accounting for <2% of the reads compared to the FL and W fractions in which the *Actinobacteria* were the second most abundant phylum, accounting for nearly a third of the communities (Figure 3). Additionally, the *Actinobacteria* accounted for a significantly greater proportion of the LE than LA microbiomes (∼2 log_2_ fold, *p* < 0.05; Supplementary Figure 6).

The *Proteobacteria* (>40%) and *Bacteroidetes* were also among the dominant phyla in all LE samples (Figure 3), however in contrast to LA the *Bacteroidetes* was significantly more abundant in the colonies (*p* < 0.01; Supplementary Figure 6), accounting for 31% of the reads compared to ∼15% in the FL and W fractions. Similar to LA, the γ-*proteobacteria* were the most abundant proteobacteria class (∼50%) and significantly enriched (*p* < 0.05; Supplementary Table 7) in the MCA fraction being primarily composed of the *Burkholderiaceae* (11.7% of total) and *Nitrosomonadaceae* (5.7% of total) families. The α*-proteobacteria*, however, were the primary class in the FL and W fractions (>50%), belonging almost exclusively to the *SAR11* clade, but were significantly depleted in the MCA fraction (∼40%, *p* < 0.01; Supplementary Table 7) consisting of member of the *Caulobacterales*, *Sphingomonadales*, *Acetobacterales*, and *Rhodobacterales* orders. Among the *Bacteroidetes*, the MCA communities were significantly enriched in the *Cytophagales* order (*p* < 0.001; Supplementary Table 7) and significantly depleted in the *Chitinophagales* and *Sphingobacteriales* orders (*p* < 0.001, Supplementary Table 7). Among the eight other significantly differentially abundant phyla among the fractions (*p* < 0.05; Supplementary Figure 6), several exhibited large fold changes. Notably, the *Verrucomicrobia* were ∼3 log_2_ fold less abundant in the MCA fraction, while *Chloroflexi* exhibited the largest differential abundance, being > 5 log_2_ fold less abundant in the MCA fraction (*p* < 0.001; Supplementary Figure 6). Conversely, the *Firmicutes* phylum was significantly enriched in the MCA fraction (*p* < 0.05; Supplementary Figure 6), in which it was the fourth most abundant phyla accounting for 4.0% of the communities compared to <2.0% in the FL and W communities (Figure 3). Across lakes, the LE bacterial microbiomes were less diverse than LA communities, with significantly fewer ASVs in the FL and MCA fractions and significantly lower Shannon richness and Pielous evenness in the FL and W fractions (Supplementary Figure 4 and Supplementary Table 5).

### Spatial-Temporal Dynamics of Bacterial Microbiomes Relative to Environmental Conditions

**FIGURE 6:**
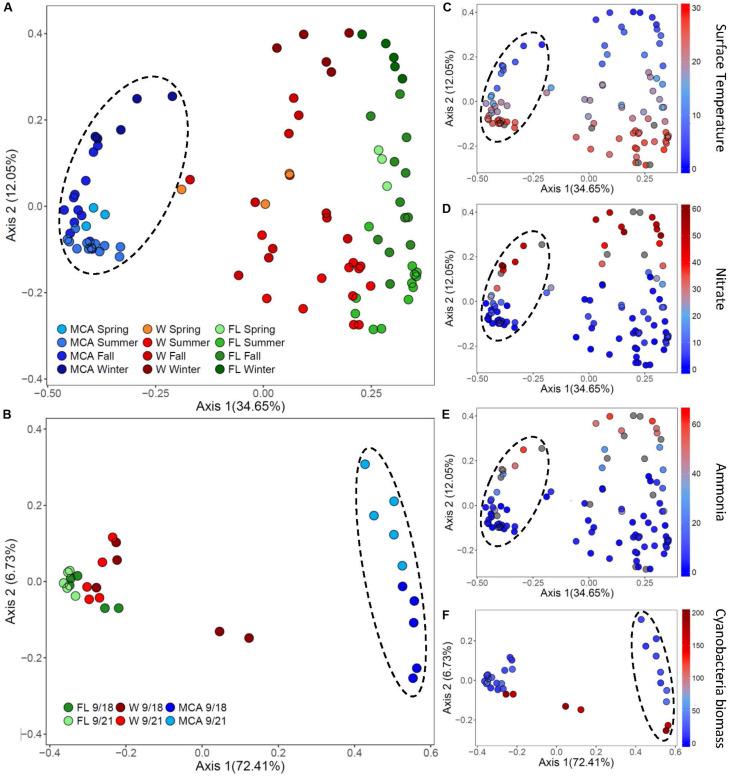
Principal coordinates analysis (PCoA) conducted on ASV abundances showing the dissimilarity of heterotrophic bacteria compositions between samples in **(A)** Lake Agawam and **(B)** Lake Erie. Color denotes the sample size fraction (MCA, FL, and W) and the color gradient indicates season in Lake Agawam and transect in Lake Erie. Percent’s listed on the axes represents the percent variation explained. **(C–E)** Show the environmental parameters that were significantly correlated to community dissimilarity, with color gradient indication environmental parameter value. **(C–E)** Represent Lake Agawam and **(F)** represents Lake Erie. Circles indicate significant clustering of the MCA communities.

The composition of the bacterial microbiomes was more variable temporally than spatially during the *Microcystis* blooms, with the LA community dissimilarities in all three size fractions being significantly (*p* < 0.05) more dispersed than the LE communities (Figure 6, Supplementary Table 3, and Supplementary Figure 5). In LA, temporal variability accounted for 12% of the variance among samples per fraction (PCOA axis 2) associated with similar seasonal changes in all three fractions, whereas site only accounted for about 7% variance (PCoA axis 2) in LE (Figure 6). Within LA, the MCA communities were the least variable over time, exhibiting significantly greater homogeneity (*p* < 0.01) across samples than the FL and W fractions (Supplementary Table 3). In contrast, within LE the MCA communities were the most dispersed over space and time of the three fractions and were significantly (*p* < 0.01) more dispersed than the free-living microbiomes (Figure 6 and Supplementary Table 3).

The beta diversity of the LA communities in all fractions was significantly (*p* < 0.01) correlated with temperature and N pools (Supplementary Table 8) with spring/summer communities associated with warmer temperatures and lower nitrate and ammonium concentrations while the fall/winter communities were associated with colder temperatures and higher nitrate and ammonium availability (Figure 6). In all fractions, Bray Curtis dissimilarities were most strongly correlated with temperature (ρ = 0.54) followed by nitrate (ρ = 0.32) and then ammonium (ρ = 0.26), with the strongest association between the MCA fraction compositions and these parameters (Supplementary Table 8). The alpha diversity of the LA bacteria assemblages was more strongly associated with nutrient conditions, however, as the richness indices of all three size fractions were significantly correlated with nitrate and ammonium, but significantly anticorrelated with temperature in the FL and W fractions (Supplementary Table 6). The alpha diversity of the MCA bacteria communities was significantly anticorrelated to cyanobacteria abundance whereas the diversity of the FL and W communities significantly increased with increasing cyanobacteria biomass (Supplemental Table 6).

Within each of the LA size fractions, there were distinct groups of covarying ASVs, known as balances, that were significantly different in abundance between months associated with four temperature/nitrate regimes; a low nitrate/moderate temperature (<1 μM, 17–28°C) group from May to June, a low nitrate/high temperature (<5 μM, 22–28°C) group from June to September, a moderate nitrate and temperature (0.09 – 40 μM, 7–24°C) group from September to November and a high nitrate/low temperature (18 – 58μM, <15°C) group from November to January (Supplementary Figure 7). The abundances of these groups were most significantly correlated with temperature, followed by nitrate, and then ammonium (Supplementary Figure 7), together explaining approximately 25% of the variability within the size fractions. At coarser taxonomic levels, there were notable seasonal changes in the relative abundance of the *Gemmatimonadetes* phylum in the MCA fraction, which made up a greater proportion of the community during the fall bloom from September to October, as did members of the *Anaerolineae* (*Chloroflexi*) class during this time (Figure 2). Additionally, in the MCA fraction, the *Bacteroidetes* phylum (primarily *Cytophagales*) and members of the *Rhizobiales* order were more abundant during the spring while the *Bdellovibrionales* (δ*-proteobacteria*), *Spirochaetes*, *Bacilli* (*Firmicutes*) and *Sphingobacteriales* were most prominent during periods of bloom demise in the summer and winter (Figure 2).

In LE, there were no clear spatial compositional (beta diversity) patterns within the size fractions, although there was an obvious separation among the MCA communities between transect dates (Figure 6). The shift in the MCA and W communities Bray Curtis dissimilarities were significantly correlated with cyanobacterial abundance (*p* < 0.05, Supplementary Table 8 and Figure 6); notably the communities from sites M2 and M3 on September 18th were associated with high cyanobacteria levels and were distinct from all other samples within the respective size fractions where there were lower cyanobacterial levels including the same sites sampled three days later (Figure 6). The community richness in all three fractions was significantly correlated with ammonium levels (*p* < 0.05; Supplementary Table 6) and the richness of the LE MCA communities was significantly anticorrelated with cyanobacteria abundance (*p* < 0.05; Supplementary Table 6). Patterns of co-varying taxa were associated with differences in cyanobacteria abundance and were strongest within the MCA fraction (Supplementary Figure 8). Specifically, members of the *Sphingomonadaceae* and *Burkholderiaceae* families were more prominent in the MCA fraction in samples with low cyanobacteria abundances while members of the *Microscillaceae* and *Nitrosomonadaceae* families were more abundant in the MCA and FL fractions in samples with high cyanobacteria abundance (M2 and M3 on September 18th; Supplementary Figure 9).

### Potential Functional Groups of Heterotrophic Bacteria

**FIGURE 7:**
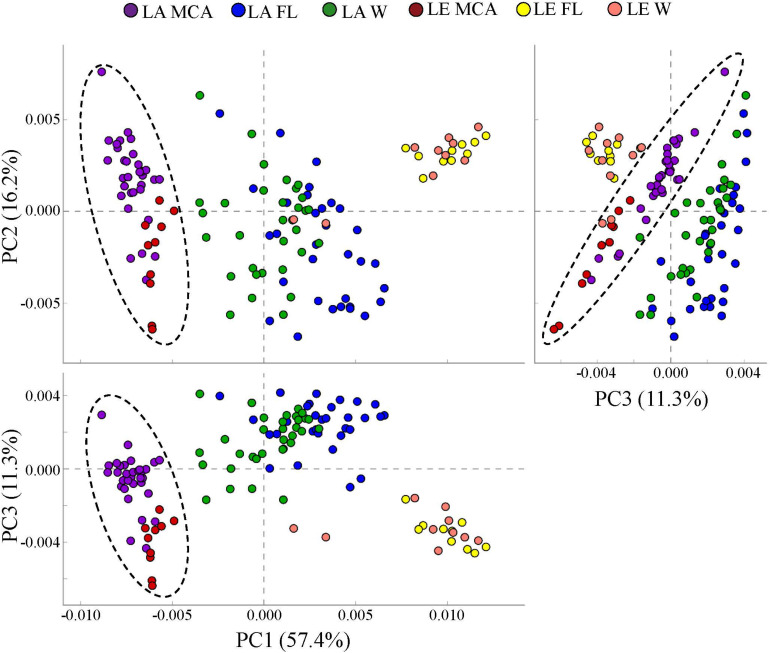
Principal coordinates analysis (PCoA) of KEGG ortholog (KOs) abundances determined via PICRUSt analysis showing the dissimilarity of predicted metagenomes of the heterotrophic bacteria communities in the MCA, FL, and W water fractions of Lake Agawam and Lake Erie. Circles indicate significant clustering of the MCA communities. Percent’s listed on the axes represents the percent variation explained.

PICRUSt analysis predicted that the MCA microbiomes possessed functional metagenomes that were significantly (*p* < 0.05; PERMANOVA) distinct from the FL and W fractions in both lakes (Figure 7). Differences between MCA and other size fractions were predicted to include genes involved in alkaline phosphatase activity, nitrogen cycling, and microcystin degradation. Despite taxonomic differences between the lakes, the predicted metagenomes of the MCA fractions in Lake Erie and Lake Agawam clustered into a statistically indistinguishable group suggesting the communities shared a similar functional potential within this fraction (Figure 7). In contrast, the FL and W predicted metagenomes, while similar within lakes, formed distinct clusters between lakes (Figure 7).

**FIGURE 8:**
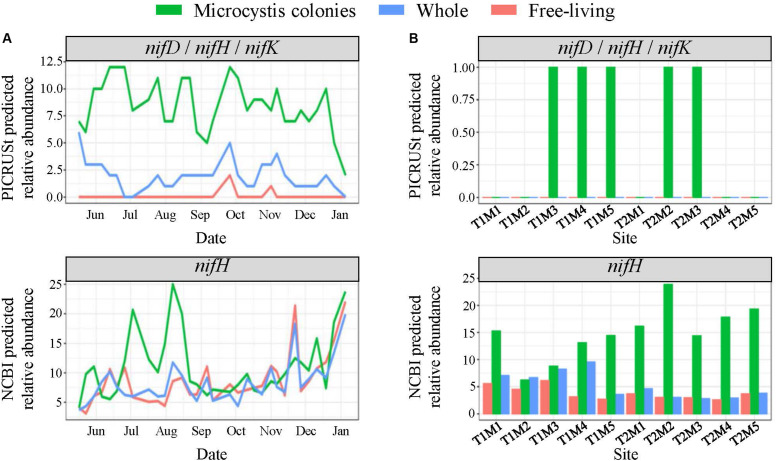
PICRUSt and NCBI predicted total relative abundances of the *nif* nitrogen fixation genes **(A)** throughout the time series in Lake Agawam and **(B)** across space in Lake Erie in the MCA, FL, and W fractions. Relative abundances quantified from predicted metagenomes generated using PICRUSt analysis with the KEGG genome database represent the *nifD* (K02586), *nifH* (K02588) and *nifK* (K02591) genes which all exhibited same pattern of abundance. The NCBI predicted abundances represent the total relative abundance of taxa with *nifH* sequences in the NCBI database which is a larger database for genes of interest than in the KEGG database.

In LA, among the predicted phosphatase genes, the *phoD* gene was ∼20% more abundant in the MCA fraction with the highest abundances coinciding with the late summer bloom, while the *phoA/B* gene was about two-fold more prevalent in the FL and W fractions, peaking in the spring (Supplementary Figure 10). The MCA fraction exhibited the highest predicted abundances of genes involved in N cycling. Specifically, the *nifD*, *nifH*, and *nifK* genes involved in N_2_-fixation were significantly enriched in the MCA fraction (*p* < 0.05), in which they were three-to-four-fold more abundant than the FL and W fractions throughout the time series (Figure 8). Similarly, the relative abundance of taxa with available *nifH* sequences in the NCBI database were two-to-three-fold higher in the MCA fraction and from July to mid-August coinciding with the lowest combined nitrate and ammonium concentrations (Figure 8). Several genes involved in the assimilatory- and dissimilarity- nitrate reduction pathways and the denitrification pathway were predicted to be more abundant in the MCA microbiomes (Supplementary Figure 10). Finally, the predicated relative abundance of potential microcystin-degrading bacteria was one and a half-to-two-fold more abundant in MCA (Supplementary Figure 10) and was inversely correlated with microcystin concentrations (Table 1) and cyanobacterial abundance (Figure 2).

In LE, all phosphatase genes were predicted to be at up to 50% higher abundances in the MCA microbiomes in all samples except sites M2 and M3 on September 18th (Supplementary Figure 11). The relative abundance of the *phoA/B* and *phoD* genes in the MCA fraction tended to parallel cyanobacteria abundance while the *phoX* gene was more abundant during the September 21st transect when cyanobacteria levels were lower (Supplementary Figure 11). The relative abundance of taxa with *nifH* sequences in the NCBI database accounted for ∼15–25% of the MCA communities compared to less than 5% in the FL and W fractions, with higher abundances associated with lower cyanobacteria levels, particularly in the September 21st transect (Figure 8). Genes associated with the denitrification, dissimilatory and assimilatory nitrate reduction pathways were also more abundant within colonies apart from the *nrfA* and *nrfH* genes (denitrification) that were more abundant in the FL fraction (Supplementary Figure 11). Microcystin-degrading bacteria were more abundant in the MCA fraction on September 21st and peaked at site M2 on the 21st where microcystin concentrations were lowest (Supplementary Figure 11 and Table 1).

## Discussion

During this study, bacterial microbiomes associated with *Microcystis* colonies were significantly different from the free-living and whole water populations in terms of phylogenetic composition, diversity, and potential functionality. Size fractionation explained the largest amount of variability in the composition of the bacterial communities, accounting for over a quarter of the variation among both lakes and up to 75% of the variation within individual lakes. Such dissimilarity is consistent with prior studies that have identified compositionally different free-living and cyanobacteria-associated bacteria assemblages in lake systems (Shi et al., 2012; Parveen et al., 2013; Akins et al., 2018). The present study, however, further demonstrates differences even between the whole bacterial community and colony-associated communities. Moreover, this study, which to our knowledge investigated the largest number of samples and longest time series of *Microcystis* microbiomes to date, found that community dissimilarities persisted across large temporal and spatial scales and over a wide range of environmental conditions (e.g., temperature, nutrient levels, bloom intensity). While LA and LE had different microbiomes, likely due to differing indigenous populations, there were shared taxonomic characteristics among each fraction across lakes with samples from the same fraction clustering together. While cross lake comparisons of free-living and cyanobacteria-associated bacteria have been previously conducted (Akins et al., 2018), this study demonstrates the conserved similarities among fractions across a broader geographical region in lakes with highly different hydrodynamic, physical, and chemical properties. Moreover, despite taxonomic differences in total bacterial assemblages across lakes, the LA and LE MCA communities exhibited similar predicted metagenomes, suggesting conserved functional characteristics among *Microcystis*-associated communities. These significant and consistent differences in taxonomic composition compared to the other fractions along with the conserved predicted functionality among the MCA microbial communities together suggest that *Microcystis* colonies harbor select bacteria with distinct metabolic capabilities.

There was evidence of selective pressure on the microbiomes associated with *Microcystis* colonies. In both LA and LE, the MCA communities were the least diverse of the three fractions examined in terms of ASV richness, a trend also observed in studies by Shi et al. (2012) and Yang et al. (2017) in Chinese lakes. Further, the MCA alpha diversity was significantly anti-correlated with cyanobacteria abundance unlike the FL and W communities, with a notable divergence in taxonomic composition among high biomass sites in LE, suggesting that the diversity of MCA microbiome declines as blooms intensify. This reduction may be due, in part, to differences in the ecology of bacterial groups such as the predominantly free-living *Actinobacteria* (Kolmonen et al., 2004), which accounted for one of the largest differences between the MCA and FL/W microbiomes. While *Actinobacteria* are often the most abundant bacteria phylum in lakes, regularly accounting for over half of bacteria present within the epilimnion (Glöckner et al., 2000; Newton et al., 2011), this phylum (predominantly the *Frankiales* order) was significantly depleted in all MCA samples, constituting < 1% of the *Microcystis*-associated microbiomes; a pattern which has been reported previously (Parveen et al., 2013; Ghai et al., 2014). Additionally, *Actinobacteria* can generate supplemental light-derived energy from actinorhodopsins (Sharma et al., 2009; Newton et al., 2011), reducing their dependency on algal-derived compounds, which is a common driver for the close association between heterotrophic bacteria and phytoplankton (Paerl, 1984; Jiang et al., 2007; Briand et al., 2016).

Beyond general ecological differences that could contribute to the distinct MCA microbiomes there was also evidence of *Microcystis*-specific bacterial associations. There were significant differences in heterotrophic bacteria among the MCA and W fractions, despite having statistically identical cyanobacterial compositions, suggesting bacteria were specifically included and excluded from *Microcystis* colonies. Further, in both lakes studied here, the MCA microbiomes contained hundreds of unique ASVs (770 LA, 170 LE) not present in other fractions and more unique core ASVs compared to the FL and W fractions, suggesting that the MCA microbiomes were not simply a subset of the water column bacterial assemblages but rather contained select bacteria uniquely associated with *Microcystis*. While the present study did not directly compare the MCA microbiome to that of other large particles and cyanobacteria Shi et al. (2012) found *Microcystis* colonies harbored bacteria assemblages distinct from other large particles in the water column and Bagatini et al. (2014) identified host-specific differences in heterotrophic bacteria colonizing different cyanobacteria genera. These findings collectively suggest that differences between the size fractions were due to *Microcystis*-specific associations and selection and not differences between particle-attached and free-living bacterial communities.

Selection for specific bacteria may result from the unique mucilaginous colonial structure of naturally occurring *Microcystis*, that represents a rich source of labile organic carbon (Zhao et al., 2017) and perhaps other growth factors such as B-vitamins that collectively promote the growth of associated heterotrophic bacteria (Gibson and Smith, 1982; Croft et al., 2005; Li et al., 2018). This colonial structure also likely provides MCA-bacteria a refuge from predation (Casamatta and Wickstrom, 2000; Dziallas and Grossart, 2012). Further selection may occur through the release of bioactive compounds, as *Microcystis* and other cyanobacteria have been shown to have the ability to alter prokaryotic community composition through allelopathic interactions (Casamatta and Wickstrom, 2000; Chia et al., 2018). In a chemotaxis study by Casamatta and Wickstrom (2000), *Microcystis* associated bacteria were found to not only exhibit greater antibiotic resistance and growth yield than non-associated assemblages, but also moved toward *Microcystis* exudates while non-associated bacteria moved away compared to the control. The distinct composition of MCA microbiomes suggests that the physical-chemical environment of *Microcystis* colonies exert a selective pressure on the associated microbiomes, promoting the net growth of specific groups of bacteria.

### Environmental Drivers of Diversity and Temporal-Spatial Variation

During this study, while size fraction accounted for the largest amount of variation in taxonomic composition among the bacterial consortia within each lake, temporal and spatial fluctuations in environmental conditions were the next best explanatory variables. In regard to beta dissimilarities, there was a greater degree of temporal variation among the LA heterotrophic bacteria communities associated with abiotic factors than spatial variation among the LE communities associated with bloom intensity. These findings suggest that microbiomes are relatively similar across a bloom at a given time, even despite variations in site characteristics across a large system such as Lake Erie, but shift over time driven by larger seasonal fluctuations in temperature and nutrient availability. It is important to note, however, that our study only covered two dates in Lake Erie and one site in Lake Agawam. Future studies examining several sites over a longer duration within one or more lakes are needed to further clarify spatial-temporal relationships of microbiomes.

PCoA analyses revealed temperature was the main driver of the seasonal variance in microbial communities in LA. As a key factor regulating metabolic activity and bacterial growth (Adams et al., 2010), temperature changes can induce shifts in bacterial communities due to differing thermal niches among bacteria (Lindström et al., 2005; Shi et al., 2011; Dziallas and Grossart, 2012). However the LA time series, which was over three times as long as previously reported time series of *Microcystis*-microbiomes (Maruyama et al., 2003; Shi et al., 2011; Parveen et al., 2013) and captured a >25°C change in temperature, demonstrated the MCA microbiome was more stable over time than the FL and W communities (NMDS), with divergence only in December and January. This may perhaps reflect a buffering capacity of the mucilaginous colonial structure against exogenous environmental conditions or may perhaps indicate that other mechanisms, such as increased organic carbon availability, grazing protection, or bioactive compounds support the continued growth of select bacteria within the colonies. The divergence in the MCA fraction in the winter months from spring, summer, and fall samples may reflect temperature-induced reduction in *Microcystis* densities and bacterial growth as surface water temperatures decreased to <5°C. Further, this community shift could be a result of *Microcystis* inhibition as Shi et al. (2011) reported lowered mucilage bacteria levels in *Microcystis* colonies at low temperatures (4°C) due to increased resistance of *Microcystis* to bacterial colonization as a mechanism to survive cold winters.

Inorganic nitrogen concentrations (nitrate and ammonium) also appeared to shape MCA microbiomes during this study as the variation in these pools was significantly correlated to the alpha and beta diversity of all three fractions in the LA timeseries with the strongest association with the MCA fraction. The nitrate pool was highly anticorrelated with temperature in LA, and thus this seasonal pattern may partly reflect greater nitrate availability in the winter due to rapid uptake during warmer months (Mulholland et al., 2002; Gobler et al., 2016). However, the microbiomes also displayed statistical associations with ammonium which was less related to temperature. Further, the relative abundance of genes associated with nitrogen fixation (*nifH*) as well as potentially diazotrophic taxa peaked when N concentrations were lowest, suggesting that MCA microbiomes in general, and potentially diazotrophic taxa in particular, were partly shaped by exogenous N levels. In Lake Erie, bacterial communities were not tracked during the transition from N-replete to N-deplete conditions during late summer (Chaffin et al., 2013, 2014; Stow et al., 2015). However, our findings from LA suggest there is likely to be compositional shifts among bacterial communities, especially within the MCA fraction, associated with such changes in N availability. Berry et al. (2017) studied bacterial communities in Lake Erie from June through October and found that conductivity, which is impacted by nitrate availability, was the third best predictor of changes in total bacterial composition. Further, a study by Akins et al. (2018) noted greater divergence among free-living and cyanobacteria-associated bacterial communities as a bloom progressed from August into September. Collectively, this and prior studies support the hypothesis that the abundance of specific bacterial taxa, including diazotrophs, may be partly controlled by levels of exogenous nutrient levels.

In contrast to LA, the microbial populations were relatively consistent across the >40 km surveyed in LE, perhaps partly due to the small variation in temperature across sites. While there is limited knowledge of microbial communities across LE (Wilhelm et al., 2006), our findings are consistent with Mou et al. (2013a) who found low spatial variation across blooms in western Lake Erie with notable differences only associated with shifts in the dominant cyanobacteria, from *Microcystis*-dominated blooms in Maumee Bay to *Planktothrix*-dominated in Sandusky Bay. This spatial similarity may be linked to similar habitat complexity (Berry et al., 2017) or differential selective pressure (Chia et al., 2018) provided by the cyanobacteria. While there was relatively low variation among the LE samples, there were taxonomic shifts associated with bloom intensity, particularly within the MCA fraction. Berry et al. (2017) noted cyanobacterial blooms were a disturbance to total planktonic bacterial communities in LE, with bloom intensity and the resulting increase in pH derived from photosynthetic activity being primary explanatory variables of bacterial composition. pH is a well-known driver of bacterial growth and subsequently can result in community shifts (Lindström et al., 2005; Fierer and Jackson, 2006; Berry et al., 2017). While Berry et al. (2017) only examined the total planktonic bacterial community, the present study further resolves this relationship indicating that the free-living and even the total bacterial community are not as impacted by the bloom intensity as the MCA fraction was. This may be due to increased habitat availability with favorable pH conditions provided by the *Microcystis* colonies as blooms intensify (Dziallas and Grossart, 2012). In sum, the MCA microbiomes were least impacted by environmental conditions in the water column and most impacted by bloom intensity, further supporting the hypothesis that *Microcystis* colonies provide a favorable environment for select bacterial groups. While this study examined the effects of temperature, N, P, phytoplankton diversity and biomass, *Microcystis*, and microcystin abundance as drivers of bacterial community composition, other factors not examined here are also likely to play an important role in be shaping microbial communities.

### Conserved Functionality in MCA Microbiomes

The whole, free-living, and *Microcystis*-colony associated bacteria communities identified here had significantly different predicted metagenomes, suggesting differences in metabolic and functional capabilities. This finding was expected due to the significant differences in their phylogenetic composition and is consistent with previous studies that examined specific metabolic pathways among microbes during *Microcystis* blooms (Worm and Søndergaard, 1998). However, in stark contrast to the FL and W communities whose predicted metagenomes differed across lakes in accordance with their phylogenetic dissimilarities, the LA and LE MCA microbiomes were found to have highly similar predicted metagenomes despite significantly different taxonomic compositions. This finding evidences a conserved metabolic functionality among microbiomes harbored by *Microcystis* colonies. Although these results were generated from predictive software, they build on an emerging collection of findings from metagenomic studies describing relatively constant functional potentials of *Microcystis* microbiomes despite dynamic phylogenetic compositions (Burke et al., 2011; Steffen et al., 2012; Li et al., 2018), highlighting the importance of considering functional potential in parallel with taxonomic composition. The conserved functional potential in the MCA fraction, along with the relative taxonomic similarity over time in the LA MCA microbiomes, suggests that the stable micro-environment within *Microcystis* colonies may exert a selection pressure facilitating the growth of bacteria with distinct functions, rather than specific taxa. In contrast, FL and W communities are likely more dynamic due to greater exposure to environmental variance. In support of this hypothesis, Yang et al. (2017) found *Microcystis*-associated bacteria had highly similar community structures that formed tight networks of positive connections with *Microcystis*, while free living bacteria formed less connected and more dynamic networks. Among the biochemical pathways found to be enriched in the MCA predicted metagenomes studied here were genes associated with the transformation of N and P. In most cases, functional differences revealed via PICRUSt analysis were affirmed via manual searches for taxa with known sequences of the associated genes in NCBI databases. This enrichment in N and P cycling genes among the MCA heterotrophic bacteria is notable as it provides evidence that the colony associated bacteria may play a role in the ability of *Microcystis* to persist in changing environments as these nutrients are known drivers of *Microcystis* proliferation (Van de Waal et al., 2009; Gobler et al., 2016; Burford et al., 2019).

#### Nitrogen Fixation and N Cycling

As a non-diazotrophic cyanobacteria, *Microcystis* is reliant on exogenous N sources for growth. While *Microcystis* blooms have been associated with excessive N loading (Gobler et al., 2016) they are also known to persist under low N conditions (Chaffin et al., 2011; Jankowiak et al., 2019), including within LA during the present study from July to August when ammonium and nitrate concentrations were often <1 μM. Several lines of evidence suggest diazotrophic bacteria were significantly enriched within *Microcystis* colonies and may have partly facilitated the persistence of *Microcystis*. Genes involved in N_2_-fixation (*nifD,H,K*) were predicted to be present at significantly (*p* < 0.05) higher abundances in the MCA heterotrophic bacteria communities than the free-living or whole fractions. Further, the total relative abundance of taxa with *nifH* was five-fold higher in MCA than other fractions in LE, and nearly double in LA during peak abundances in the summer, coinciding with the period of lowest inorganic N concentrations. While photosynthesis by *Microcystis* likely enriches oxygen levels present in colonies during the day, the abundance of organic carbon and bacteria in colony mucilage likely creates zones of anoxia needed to facilitate diazotrophy at night (Zehr et al., 2003; Zhao et al., 2017), likely more so than within the water column. Supporting this finding, members of the *Rhizobiales* order were significantly (*p* < 0.05) enriched in the MCA communities in both lakes, a finding consistent with studies by Yang et al. (2017) and Akins et al. (2018). The *Rhizobiales* belong to the α-*proteobacteria*, a class known to contain many taxa that form symbiotic relations and perform N_2_-fixation in plants (Newton et al., 2011).

The contribution of endosymbiotic, diazotrophic bacteria to the cellular N demands of *Microcystis* blooms is unclear. Prior studies have identified taxa with known *nif* sequences in non-axenic *Microcystis* cultures (Shi et al., 2009; Zhu et al., 2016) and associated with naturally occurring *Microcystis* blooms (Akins et al., 2018). However, to our knowledge, a metagenomic study by Steffen et al. (2012) is the only study to directly quantify the presence of the *nif* genes within the *Microcystis* microbiome (>20 μm fraction), and no study has reported on the expression of *nif* genes within this fraction. Further, while N_2_-fixation by bacteria has been quantified in lakes (Keirn and Brezonik, 1971), including Lake Erie (Howard et al., 1970), no study has directly measured N_2_-fixation within *Microcystis* colonies. While PICRUSt predictions of *nif* genes have not been confirmed with direct measurements in *Microcystis* blooms, there has been consistency between predicted *nifH* gene presence, sequenced *nifH* abundances, and isotopic N_2_-fixation measurements among endosymbiotic bacterial assemblages in other systems (Lesser et al., 2018). The over-representation of *nifH*-containing bacteria in *Microcystis* colonies relative to other bacterial communities detected by multiple informatic methods and the increase in their predicted relative abundances during periods of low inorganic N levels suggests they may be an important, but largely overlooked, N source for *Microcystis* blooms, a hypothesis that certainly warrants deeper investigation.

Beyond heterotrophic bacteria, several diazotrophic cyanobacteria were found to be associated with *Microcystis* colonies. The epiphytic picoplankton *Pseudanabaena* (Agha et al., 2016) which is often capable of diazotrophy (Rippka, 1992; Acinas et al., 2009) was significantly enriched in the MCA fraction in LA (*p* < 0.05). Although *Pseudanabaena* has been previously detected within cyanobacterial blooms (Berry et al., 2017; Jankowiak et al., 2019), it has often been overlooked since is it a small filamentous picoplankton (Zwart et al., 2005; Willame et al., 2006; Acinas et al., 2009). Here, *Pseudanabaena* was a primary, and even dominant, component of cyanobacteria communities despite *Microcystis* accounting for the majority of the cyanobacteria biomass identified microscopically. While little is known about the relationship between *Microcystis* and *Pseudanabaena* (Agha et al., 2016), their abundances have been found to be highly correlated during *Microcystis* blooms (Berry et al., 2017). Due to its high abundance and diazotrophic capabilities, *Pseudanabaena* may be an important source of fixed nitrogen to *Microcystis* colonies as well as the surrounding waters. The diazotrophic cyanobacterium, *Dolichospermum*, was also present in both LA and LE, with peak abundances in LA under low N conditions during July-August. It has been proposed that the co-occurrence of diazotrophic cyanobacteria such as *Dolichospermum* may relieve N-stress during blooms as diazotrophy is a “leaky” process (Ohlendieck et al., 2000; Wetzel, 2001), and peak cyanobacteria abundances within the LE have been previously noted to coincide with peak diazotrophic abundances (Davis et al., 2015; Jankowiak et al., 2019). While *Dolichospermum* is a free-living cyanobacteria, it grows in long chains that can become entangled with the *Microcystis* colonies, accounting for its presence in the MCA fraction where it may act as an additional source of fixed nitrogen for the *Microcystis* colonies. However, further investigation is needed to confirm if fixed nitrogen by these cyanobacteria is being exchanged with *Microcystis*.

In addition to N fixation, there was evidence of enrichment of genes associated with other N cycling pathways in the MCA communities compared to the free-living and whole water fractions. Specifically, in both lakes, there was a higher predicted abundance of several genes involved in nitrate reduction and denitrification pathways. While these reactions primarily occur in anoxic environments (Conrad, 1996), these conditions may develop within colonies at night given their enrichment with bacteria and organic carbon (Zhao et al., 2017) and have likewise been associated with cyanobacteria aggregates in several studies (Cai et al., 2013; Qian et al., 2017; Song et al., 2017). Additionally, members of the *Nitrosomonadaceae* family, which is associated with the nitrification pathway, were found to be enriched in the MCA fraction in both lakes. Bacteria from this family are ammonia oxidizing bacteria that catalyze the first and often rate limiting step in the nitrification process (Prosser et al., 2014) and are more commonly associated with *Microcystis* than other cyanobacteria (Louati et al., 2015), likely favored by the high levels of dissolved oxygen within colonies during the day (Dziallas and Grossart, 2012). Together, these findings suggest *Microcystis* colonies are zones of dynamic N cycling.

#### Phosphatases and Phosphate Availability

One of the most notable differences between the MCA and FL/W heterotrophic bacteria communities was the high relative abundance of the *Gemmatimonadetes* in the MCA microbiomes in LA. While less abundant in LE MCA, this phylum has been previously noted as a dominant group among *Microcystis*-associated bacteria (Shia et al., 2010; Shi et al., 2012; Bagatini et al., 2014). The *Gemmatimonadetes* is a largely understudied phylum (Park et al., 2017) and with only three strains isolated to date (Zhang et al., 2003; DeBruyn et al., 2013; Zeng et al., 2015) their physiological characteristics are poorly understood. A study of the *Gemmatimonas aurantiaca* isolate by Zhang et al. (2003) found they are phosphate accumulating microbes. The transfer of P from *Microcystis* colony-attached bacteria to *Microcystis* cells has been demonstrated via isotope tracers (Jiang et al., 2007) and thus members of this group may act as a phosphorus source for *Microcystis* colonies, providing relief from P stress. Consistent with this hypothesis, the *Gemmatimonadetes* were most abundant in LA during the late summer bloom under low P conditions.

During this study, predicted metagenomes of bacteria revealed high abundances of genes in the *pho* regulon that encode for alkaline phosphatases in all three fractions. Alkaline phosphatases catalyze the hydrolysis of orthophosphate from organic molecules (Hoppe, 1983; Condron et al., 2005) during times of P scarcity (Vershinina and Znamenskaya, 2002). Among the *pho* genes, the *phoD, phoX*, and *phoA/B* genes were differentially abundant across the size fractions with each of these genes belonging to different phosphatase families, with different cofactors (Rodriguez et al., 2014), and hydrolyzing different substrates (Boulanger and Kantrowitz, 2003; Wu et al., 2007; Kageyama et al., 2011). The *phoD* gene was most prevalent in the MCA fraction in both lakes, accounting for ∼40% of the community and suggesting Microcystis harbors a select group of bacteria with this gene to aid in P acquisition. Supporting this, the highest abundance of the *phoD* gene in the MCA fraction coincided with the late summer bloom in LA when P concentrations were low and potentially a limiting macronutrient (Davis et al., 2009). The *phoD* gene has a lower trace metal requirement (one iron atom) than *phoA* (two zinc atoms) and *phoX* (two iron atoms), and trace metals can be a limiting element in lakes (North et al., 2007) and may be particularly scarce within the interior of Microcystis colonies. Hence, differences in bacterial pho genes may be partly related to the geochemical environment within colonies.

#### Bloom Toxicity and Bloom Decline

Bacterial mediated microcystin degradation is an important process for detoxifying cyanoHABs events in freshwater systems that has been documented in both artificial (Bourne et al., 2006; Ho et al., 2007, 2010) and natural environments (Mou et al., 2013b; Thees et al., 2019). Microcystin degradation was originally described in, and thought to be limited to, bacteria isolates belonging to the *Sphingomonadales* order (Bourne et al., 1996; Maruyama et al., 2006; Shimizu et al., 2012). In recent years, however, there have been a growing number of bacterial isolates reported with this ability identified via microcystin-amendment experiments coupled with sequencing (Mou et al., 2013b; Li et al., 2017; Krishnan and Mou, 2019; Thees et al., 2019). To date, the *mlr* gene cluster encodes the only confirmed microcystin-cleavage pathway in bacteria (Bourne et al., 1996), although there is growing evidence supporting alternative pathways including the *gst* gene cluster that encodes xenobiotic metabolism and is an established pathway for microcystin degradation in eukaryotic cells (Campos and Vasconcelos, 2010). For example, Manage et al. (2009) reported isolates identified as *Arthrobacter* spp., *Brevibacterium* sp., and *Rhodococcus* sp. were capable of degrading microcystin-LR but lacked the characterized *mlr* genes. Further, while *mlr* genes have been previously reported by metagenomic studies in lakes including Lake Erie (Steffen et al., 2012) these genes are not always detected in communities when microcystin degradation is quantified. In contrast to the findings of Steffen et al. (2012), Mou et al. (2013b) and Thees et al. (2019) found *mlr* genes were underrepresented among Lake Erie bacterial populations but there was an overrepresentation of the *gst* xenobiotic metabolism genes. The majority of studies examining *in situ* microcystin degradation in lakes have been performed with bioassays using free-living bacteria in the water column. In contrast, the current study explored the predicted abundances of *mlr* and *gst* genes among the FL, W and MCA fractions and found an overrepresentation of groups predicted to possess these genes within *Microcystis* colonies.

Potential microcystin-degrading bacteria were predicted to be twice as abundant in the MCA fraction (∼20%) compared to the FL and W fractions (∼10%) in all samples. *Microcystis* colonies likely act as a focal point for these bacteria as the molecule can be trapped in the mucilage when released by *Microcystis* cells due to its high viscosity (Maruyama et al., 2003). In both lakes, total microcystin concentrations were found to be lower when the potential microcystin-degrading bacteria were more abundant, with a significant inverse correlation in LA (*p* < 0.01), supporting their involvement in the removal of microcystins. Taxa belonging to the *Burkholderiales* order were abundant among the predicted microcystin-degrading bacteria, which has been noted as an important group among microcystin-degrading metagenomes in Lake Erie (Mou et al., 2013b). Additionally, bacteria from the *Cytophagales* order were among the most prominent potential microcystin-degrading bacteria identified in both lakes and were significantly enriched in the MCA fractions. While microcystin degradation has not been directly confirmed in members of this order, this group has been noted for their possible involvement in this process in other lakes (Manage et al., 2009; Lezcano et al., 2017) due to their xenobiotic metabolism and strong dependency on algal-derived dissolved organic matter (Maruyama et al., 2003; Lemarchand et al., 2006; Newton et al., 2011), with the capability to degrade complex macromolecules with structures similar to such as microcystins (Maruyama et al., 2003; Manage et al., 2009; Newton et al., 2011). In the present study, the potential microcystin-degrading bacteria were more abundant during periods of lower bloom intensity, an observation consistent with previous findings that microcystin-degrading bacteria are associated with periods of bloom decline (Maruyama et al., 2003; Manage et al., 2009; Shao et al., 2014), as microcystins are more likely to be released by senescent cells than actively growing cells. Potential microcystin-degrading bacteria such as the *Cytophagales* have also been shown to degrade *Microcystis* cells (Maruyama et al., 2003), which is in agreement with their enriched presence in the MCA fraction. Finally, beyond modulating bloom toxicity, microcystin-degrading bacteria may influence N cycling within the colony micro-environment as microcystins are N rich compounds (Tillett et al., 2000). There was, however, no correlation between the predicted abundance of microcystin-degrading bacteria and exogenous N availability during this study.

### The Utility of High-Throughput Sequencing and Predictive Software

Advancements in amplicon sequencing has provided significant insight into the structure of microbial communities in many environments but does not provide direct information regarding a community’s functional capabilities. Software such as PICRUSt (Langille et al., 2013) can provide metagenomic predictions and this study has highlighted the utility of such approaches for exploring relationships between *Microcystis* and its epibionts. Given that metagenomic studies have demonstrated that taxonomy alone may not be sufficient to comprehensively understand complex microbial interactions, it is important to also consider functional capabilities and to vet metagenomic predictions with direct measurements and/or other supporting information. Beyond PICRUSt, this study used NCBI gene searches and prior studies to support metabolic predictions. While metagenomes provide a greater degree of certainty regarding the functionality of microbial communities such approaches are more costly and, therefore, may provide a more limited coverage in time and space in comparison to amplicon sequencing. Further, metagenomic patterns also require confirmation as gene presence does not necessarily equate to metabolic activity. Software predictions can, therefore, be a useful tool allowing for a more extensive examination of microbial communities providing a finer resolution over larger temporal and spatial scales. In the present study, predicted metagenomes highlighted the conserved functionality of the MCA microbiome despite taxonomic differences and identified N_2_-fixation as a significantly enriched pathway in *Microcystis* epibionts, which has largely been overlooked to date. Finally, metabolic predictions can be performed on pre-existing amplicon datasets and, therefore, may be a useful tool to provide a foundation to direct future “-omic” studies.

## Conclusion

This study confirmed that *Microcystis* colonies harbor bacterial assemblages that are significantly different from the free-living and other particle-attached bacteria but also demonstrated these differences were conserved across geographically distinct blooms, over different stages of booms, and throughout large seasonal variations in environmental conditions. Further, this work highlights that, despite taxonomic differences, the MCA microbiomes had conserved predicted functional potentials. This supports the need to consider functionality in addition to taxonomy to better understand microbial interactions as more work emerges demonstrating that taxonomic variability of *Microcystis* microbiomes can diverge from their functionality. This finding also suggests *Microcystis* colonies harbor select bacteria that perform specific functions although these functions may be performed by different taxa in different systems. Key pathways predicted to be enriched in the MCA fraction included microcystin degradation and N and P cycling, with a notable enrichment of N_2_-fixation genes. This pathway has largely been overlooked in *Microcystis* colonies and warrants further investigation as diverse N assimilation pathways within colonies may contribute to the persistence of *Microcystis* blooms under unfavorable conditions. This study, therefore, may serve as a foundation to guide future “-omic” studies that can further explore such hypothetical interactions.

## Data Availability Statement

The datasets generated for this study can be found in the NCBI SRA BioProject PRJNA601166.

## Author Contributions

JJ and CG conducted the research and contributed to the writing of this manuscript.

## Conflict of Interest

The authors declare that the research was conducted in the absence of any commercial or financial relationships that could be construed as a potential conflict of interest.

## Footnotes

1 https://www.ncbi.nlm.nih.gov/sra/PRJNA601166
