# Supplementary figures and images for: The Composition and Function of Microbiomes Within Microcystis Colonies Are Significantly Different Than Native Bacterial Assemblages in Two North American Lakes
Source: Front Microbiol. 2020 May 28;11:1016. doi: 10.3389/fmicb.2020.01016 (PMC7270213; doi:10.3389/fmicb.2020.01016)

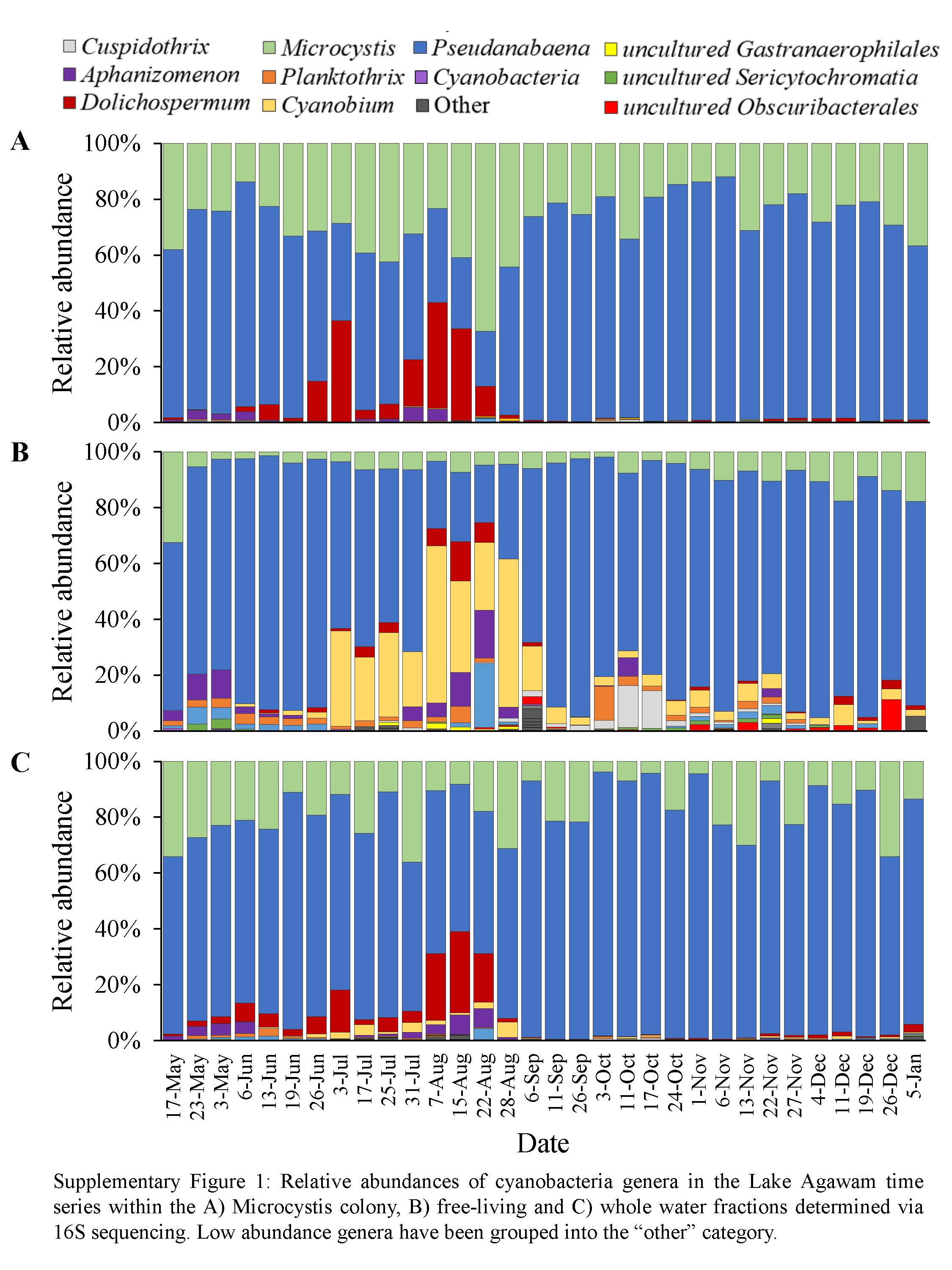

Supplement: Supplementary file 9 [file Image_1.JPEG]

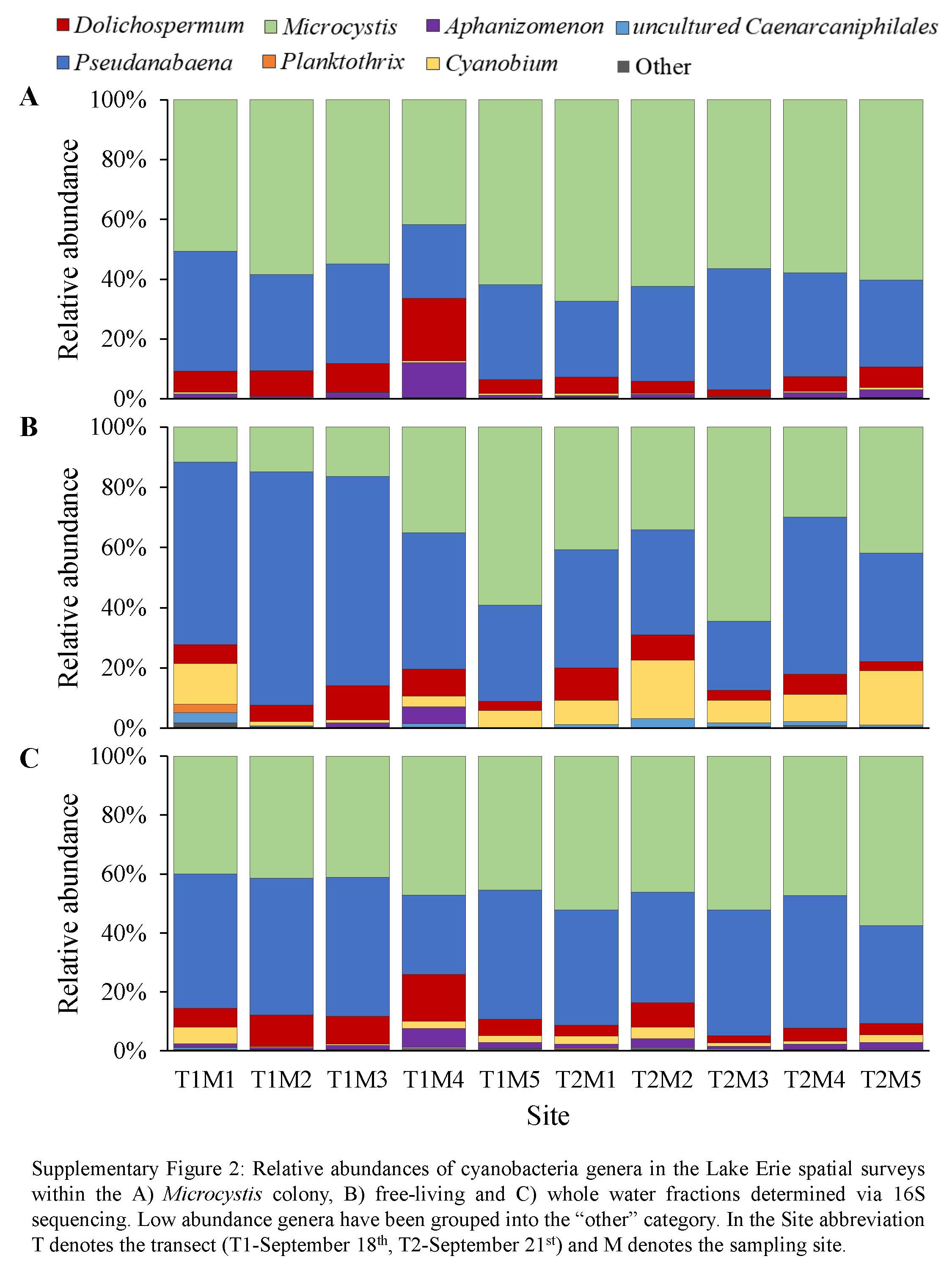

Supplement: Supplementary file 10 [file Image_2.JPEG]

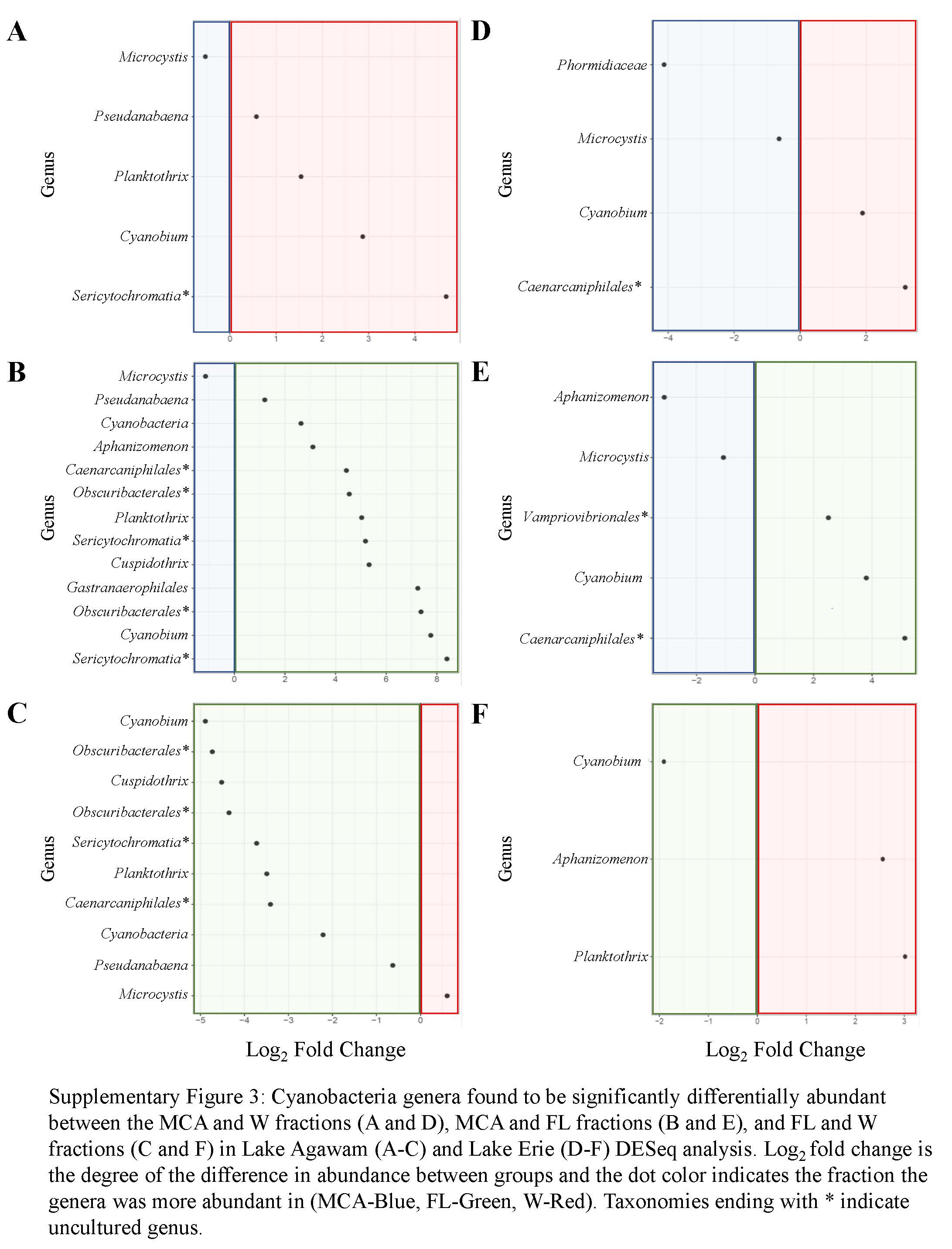

Supplement: Supplementary file 11 [file Image_3.JPEG]

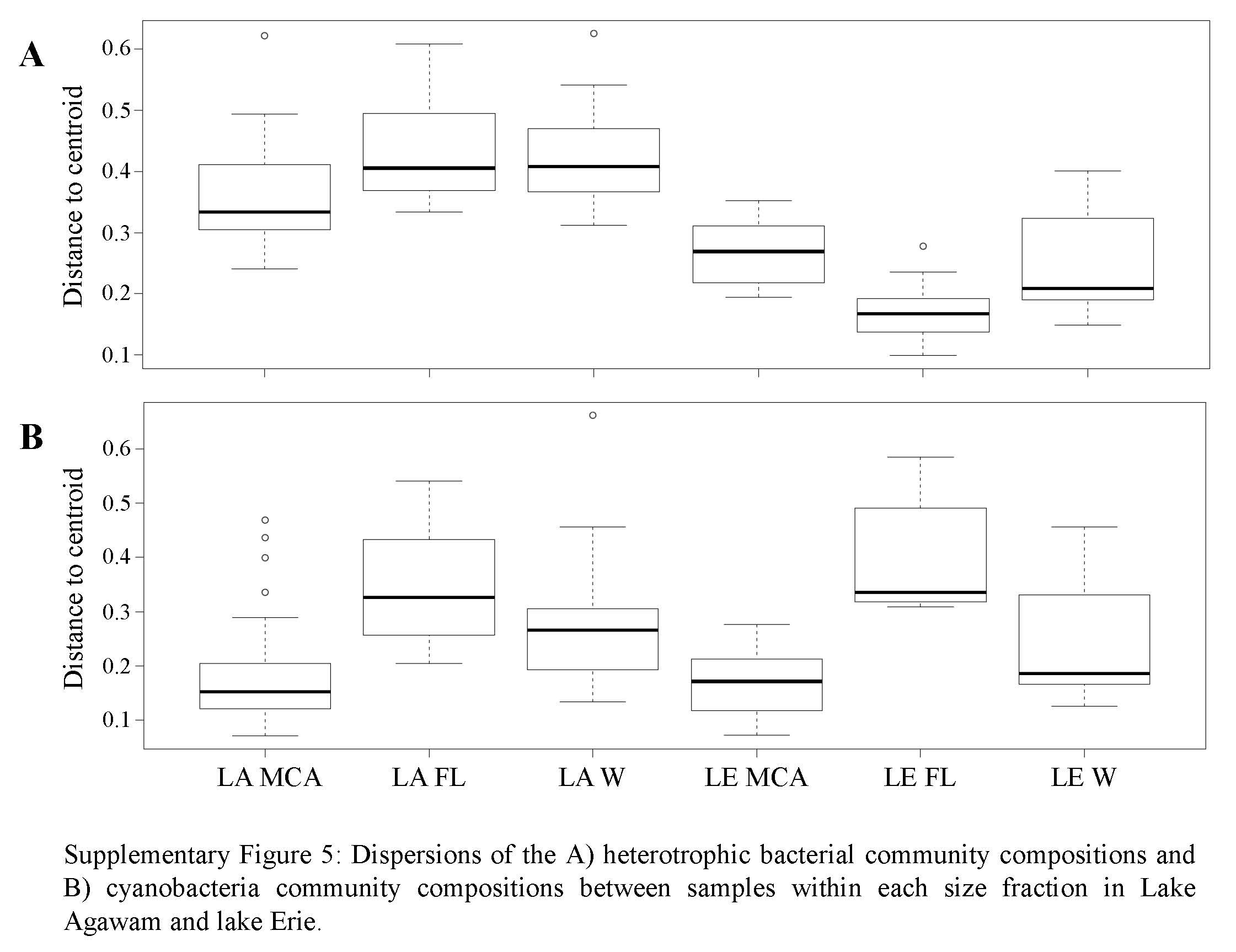

Supplement: Supplementary file 13 [file Image_5.JPEG]

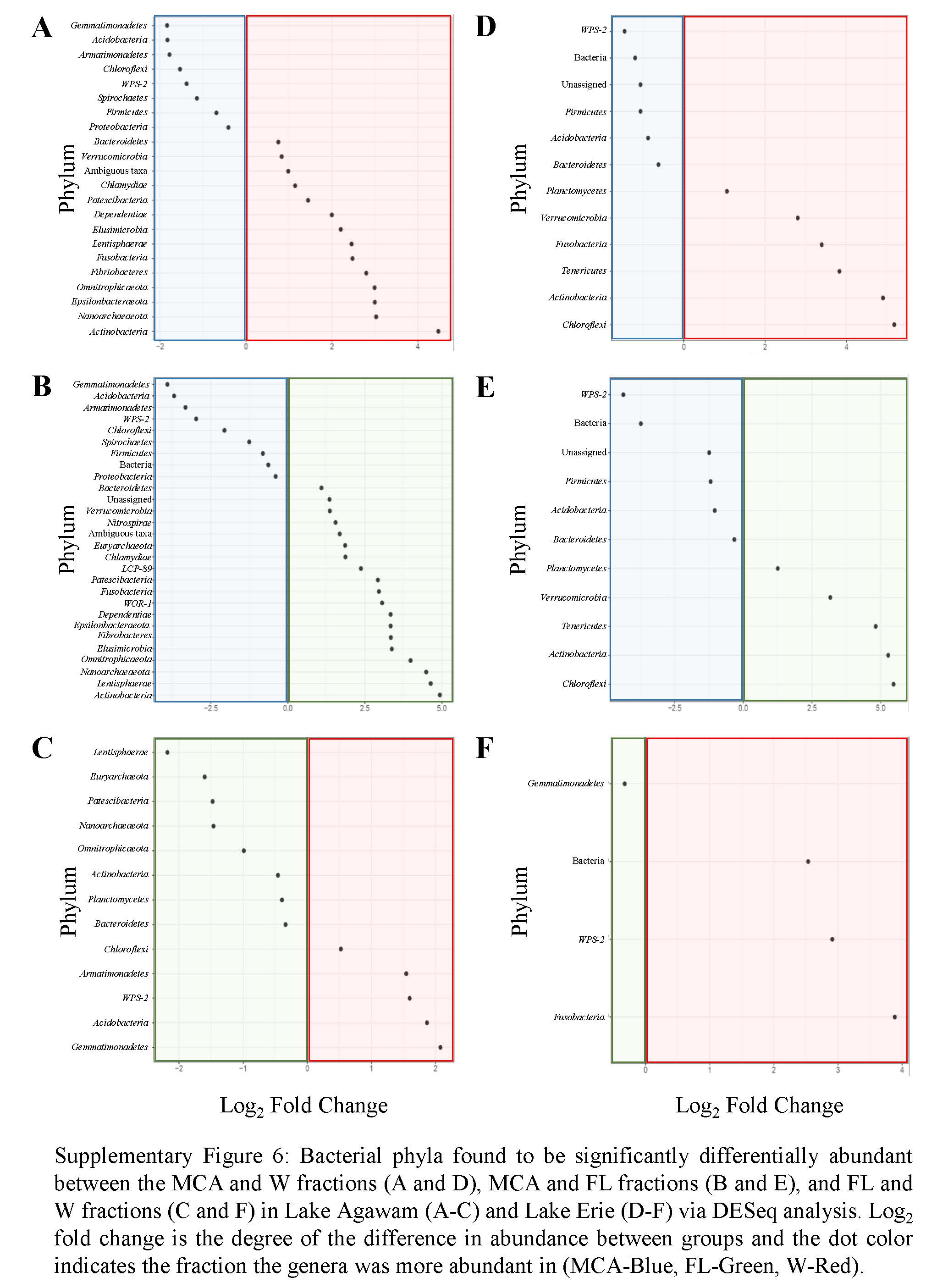

Supplement: Supplementary file 14 [file Image_6.JPEG]

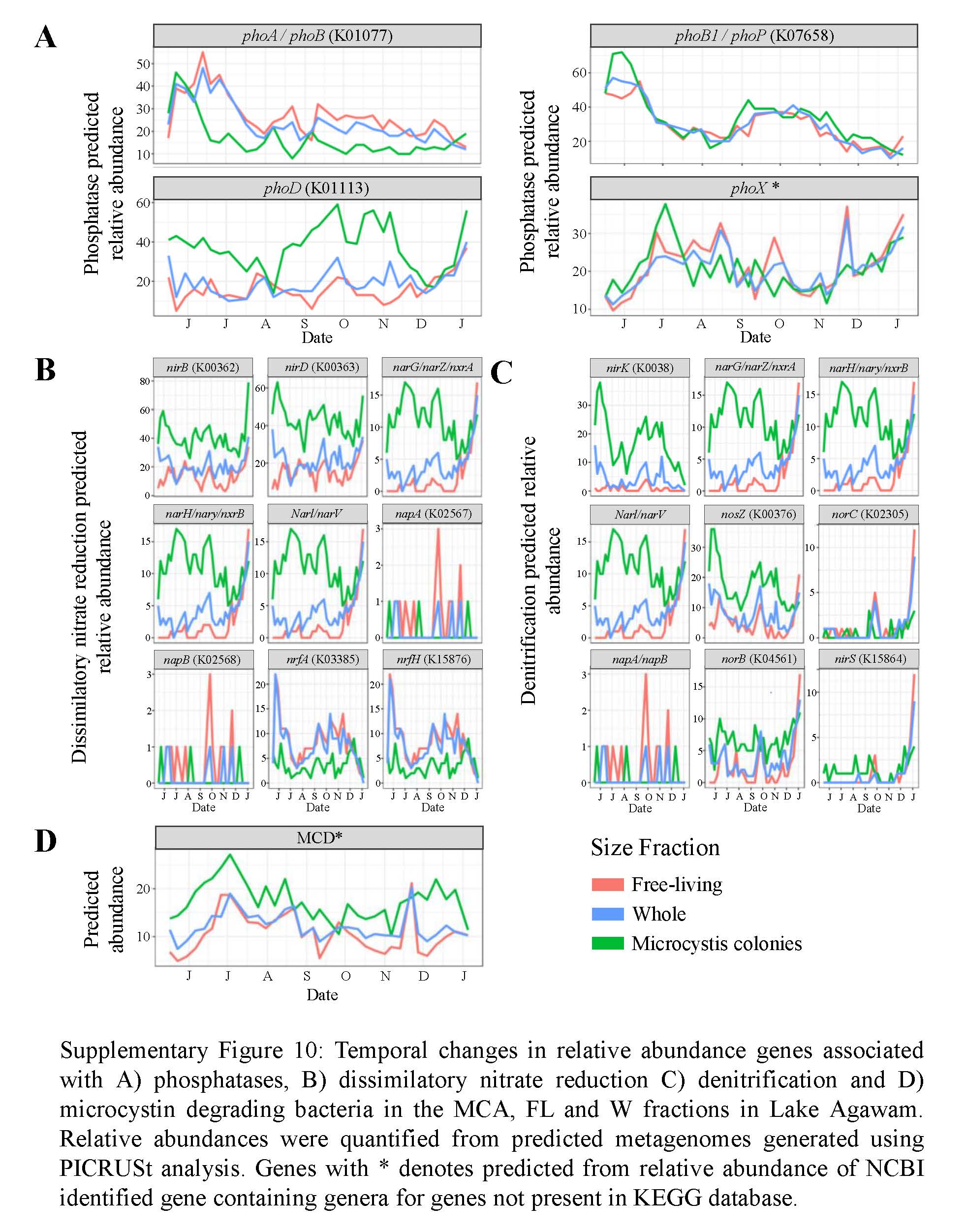

Supplement: Supplementary file 18 [file Image_10.JPEG]

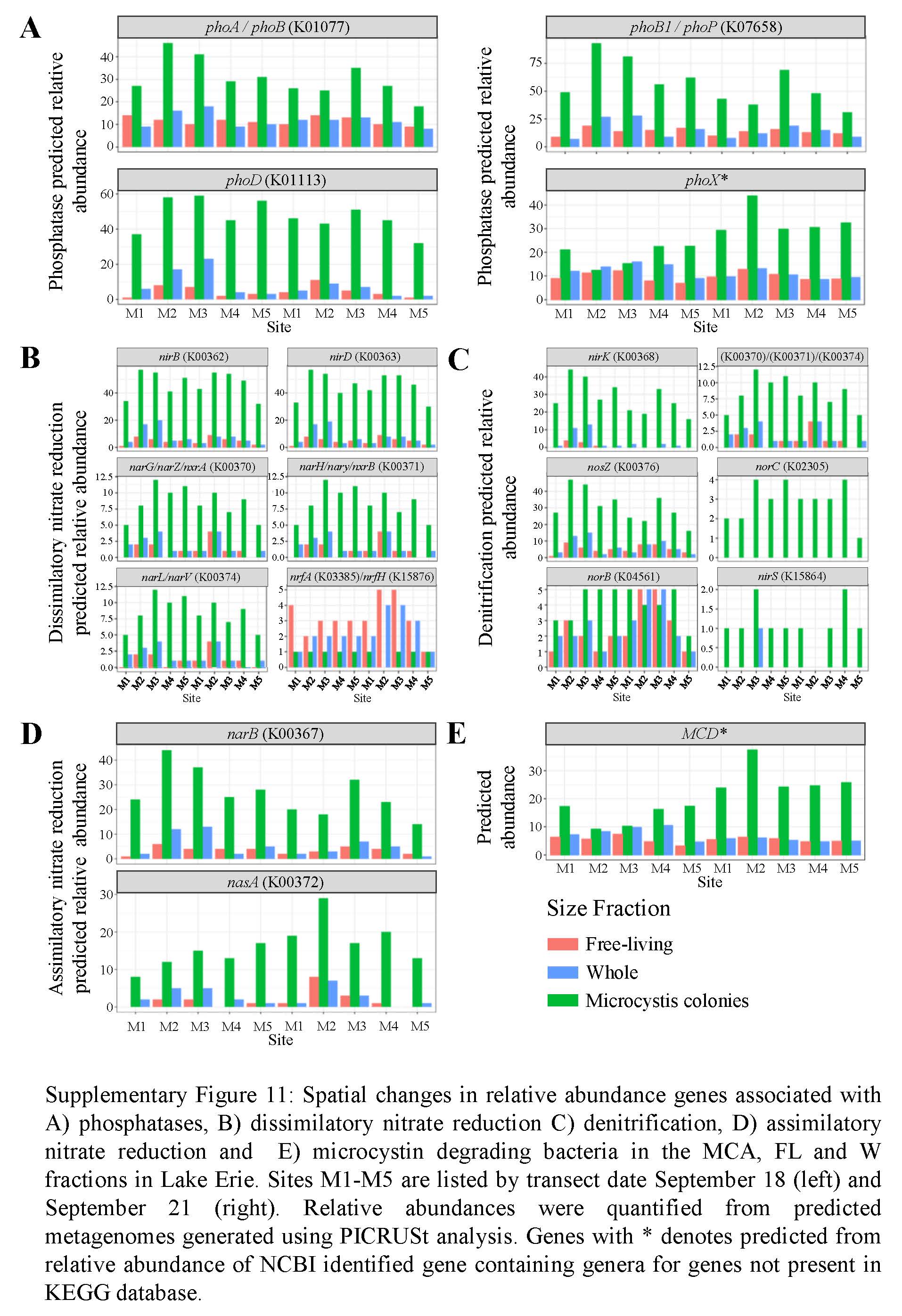

Supplement: Supplementary file 19 [file Image_11.JPEG]
